# StabJGL: a stability approach to sparsity and similarity selection in multiple-network reconstruction

**DOI:** 10.1093/bioadv/vbad185

**Published:** 2023-12-19

**Authors:** Camilla Lingjærde, Sylvia Richardson

**Affiliations:** MRC Biostatistics Unit, University of Cambridge, Cambridge CB2 0SR, United Kingdom; MRC Biostatistics Unit, University of Cambridge, Cambridge CB2 0SR, United Kingdom

## Abstract

**Motivation:**

In recent years, network models have gained prominence for their ability to capture complex associations. In statistical omics, networks can be used to model and study the functional relationships between genes, proteins, and other types of omics data. If a Gaussian graphical model is assumed, a gene association network can be determined from the non-zero entries of the inverse covariance matrix of the data. Due to the high-dimensional nature of such problems, integrative methods that leverage similarities between multiple graphical structures have become increasingly popular. The joint graphical lasso is a powerful tool for this purpose, however, the current AIC-based selection criterion used to tune the network sparsities and similarities leads to poor performance in high-dimensional settings.

**Results:**

We propose stabJGL, which equips the joint graphical lasso with a stable and well-performing penalty parameter selection approach that combines the notion of model stability with likelihood-based similarity selection. The resulting method makes the powerful joint graphical lasso available for use in omics settings, and outperforms the standard joint graphical lasso, as well as state-of-the-art joint methods, in terms of all performance measures we consider. Applying stabJGL to proteomic data from a pan-cancer study, we demonstrate the potential for novel discoveries the method brings.

**Availability and implementation:**

A user-friendly R package for stabJGL with tutorials is available on Github https://github.com/Camiling/stabJGL.

## 1 Introduction

Network models have in recent years gained great popularity in many areas. In statistical omics, networks can be used to decode aspects of unknown structures, and hence study the relationships between genes, proteins, and other types of omics data. In health data sciences, rich datasets are more and more frequently encountered, enabling the development of models integrating a variety of biological resources. In the high-dimensional setting commonly found in omics, sharing information between independent observations from data sources with shared structures—which could be different tissues, conditions or patient subgroups—can give a valuable increase in statistical power while elucidating shared biological function. A key question is how to combine the different data sources into a single model.

If a Gaussian graphical model is assumed, a conditional (in)dependence network can be estimated by determining the non-zero entries of the inverse covariance (precision) matrix of the data. With its good performance in numerical studies, the “graphical lasso” (Glasso) of Friedman *et al.* (2008) is a state-of-the-art method for precision matrix estimation in the setting of Gaussian graphical models. The method combines $L_{1}$ regularization with maximum likelihood estimation. Other notable methods include the neighborhood selection approach of Meinshausen and Bühlmann (2006) and the graphical SCAD (Fan *et al.* 2009). Notable Bayesian methods include the Bayesian Glasso (Wang 2012), Bayesian spike-and-slab approaches (Wang 2015), and the graphical horseshoe (Li *et al.* 2019a).

If multiple related datasets available, there are several ways to leverage common network structures. If focusing on one data type’s network structure, data from other types can enhance inference via weighted Glasso methods (Li and Jackson 2015, Lingjærde *et al.* 2021). However, to compare network structures across datasets, such as patient subgroups, a joint approach that leverages common information while preserving the differences can increase statistical power and provide interpretable insight.

In the area of multiple Gaussian graphical models, existing methods include the group extension of the Glasso to multiple networks of Guo *et al.* (2011), the Bayesian spike-and-slab joint graphical lasso (JGL) (Li *et al.* 2019b), and the Markov random field approach of Peterson *et al.* (2015). The widely used JGL of Danaher *et al.* (2014) extends the Glasso to a multiple-network setting and provides a powerful tool for inferring graphs with common traits. It employs two different penalty functions—group joint graphical lasso (GGL) and fused joint graphical lasso (FGL)—with the latter recommended for most applications. From this point forward, any mention of the JGL will imply the fused version, unless otherwise specified. The method needs tuning of two regularization parameters for controlling (i) the number of non-zero effects, and (ii) the similarity between networks, respectively. However, the default parameter selection routine based on the AIC (Akaike *et al.* 1973) often results in severe over-selection in high-dimensional data, potentially impacting performance negatively (Foygel and Drton 2010, Liu *et al.* 2010).

We propose a stable and well-performing penalty parameter selection method for the JGL, combining the model stability principle of Liu *et al.* (2010) with likelihood-based selection for high-dimensional data (Foygel and Drton 2010). The resulting method inherits the powerful traits of the JGL while mitigating the risk of severe under- or over-selection of edges in high-dimensional settings. We provide an R package, stabJGL (stable sparsity and similarity selection for the JGL), which implements the method.

The article is organized as follows. In Section 2, we first describe the Gaussian graphical model framework and the penalized log-likelihood problem we aim to solve. We then describe our proposed algorithm. In Section 3, we demonstrate the performance of our proposed method on simulated data and apply it proteomic data from a pan-cancer study of hormonally responsive cancers. Finally, we highlight possible extensions in Section 4.

## 2 Methods

### 2.1 Gaussian graphical models

In a gene network model, genes are represented by “nodes” and associations between them are represented by “edges.” Given measurable molecular units each corresponding to one gene (e.g. the encoded protein or mRNA), a network, or graph, can be constructed from their observed values.

Consider *n* observed values of the multivariate random vector $x={\left(X_{1},\ldots ,X_{p}\right)}^{T}$ of node attributes, with each entry corresponding to one of *p* nodes. If we assume multivariate Gaussian node attributes, with an n×p observation matrix X with i.i.d. rows $x_{1},\ldots ,x_{n}∼N(0,\Sigma )$, a “partial correlation network” can be determined by estimating the inverse covariance matrix, or precision matrix, $\Theta =\Sigma^{-1}$. Specifically, the partial correlation between nodes *i* and *j*, conditional upon the rest of the graph, is given by

$$\rho_{ij|V\backslash \{i,j\}}=-\frac{\theta_{ij}}{\sqrt{\theta_{ii}\theta_{jj}}},$$

where the $\theta_{ij}$’s are the entries of Θ and *V* the set of all node pairs (Lauritzen 1996). The partial correlations coincide with the conditional correlations in the Gaussian setting. Because correlation (resp. partial correlation) equal to zero is equivalent to independence (resp. conditional independence) for Gaussian variables, a conditional independence graph can thus be constructed by determining the non-zero entries of the precision matrix. To ensure invertibility, the precision matrix also required to be positive definite, Θ≻0.

In high-dimensional settings, the sample covariance matrix $S=\frac{1}{n-1}X^{T}X$ is rarely of full rank and thus its inverse cannot be estimated directly. It is common to assume sparse network, meaning the number of edges in the edge set *E* is small relative to the number of potential edges in the graph (i.e. the sparsity measure $2|E|/(p^{2}-p)$ is small). Penalized methods, such as the Glasso (Friedman *et al.* 2008) are well established for sparse Gaussian graphical model estimation. In the case of there being multiple (related) datasets available, such as from different tissue types, rather than estimating each network separately much statistical power could be gained by sharing information across networks through a joint approach.

### 2.2 Penalized log-likelihood problem

Assume a network inference problem with *K* groups. We let $\left\{\Theta \}=(\Theta^{\left(1\right)},\ldots ,\Theta^{\left(K\right)}\right)$ be the set of their (unknown) precision matrices, and assume that the set of $\sum_{k=1}^{K}n_{k}$ observations are independent. We aim to solve the penalized log-likelihood problem (Danaher *et al.* 2014)

(1) $$\begin{array}{c} \begin{array}{c} \left\{\hat{\Theta }\right\}=\underset{\left\{\Theta ≻0\right\}}{\text{argmax}}\{\sum_{k=1}^{K}n_{k}[\text{log}(\det(\Theta^{\left(k\right)}))-\text{tr}(S^{\left(k\right)}\Theta^{\left(k\right)})] \end{array}\\ -P(\{\Theta \})\}, \end{array}$$

where $S^{\left(k\right)}$ is the sample covariance matrix of group *k* and P(⋅) is a penalty function. In (1), det(⋅) denotes the determinant and tr(⋅) denotes the trace. The JGL employs the fused penalty function

(2) $$P(\{\Theta \})=\lambda_{1}\sum_{k=1}^{K}\sum_{i\neq j}\text{abs}(\theta_{ij}^{\left(k\right)})+\lambda_{2}\sum_{k<k^{\prime }}\left|\right|\Theta^{\left(k\right)}-\Theta^{\left(k^{\prime }\right)}|{|}_{1},$$

where $\lambda_{1}$ and $\lambda_{2}$ are positive penalty parameters, abs(⋅) denotes the absolute value function and $||\cdot |{|}_{1}$ denotes the $L_{1}$ penalty. This penalty applies $L_{1}$ penalties to each off-diagonal element of the *K* precision matrices as well as to the differences between corresponding elements of each pair of precision matrices. The parameter $\lambda_{1}$ controls the sparsity, and the similarity parameter $\lambda_{2}$ controls the degree to which the *K* precision matrices are forced toward each other, encouraging not only similar network structures but also similar precision matrix entries. This way, the dependency between observed datasets is modeled through their underlying graphical structures. It is important to note that a necessary assumption of the resulting model is that given their respective graphical structures, observations are independent across and within each dataset. The current penalty parameter selection approach for $\lambda_{1}$ and $\lambda_{2}$ is based on the AIC (Danaher *et al.* 2014), and while suitable for determining network similarities, likelihood-based selection criteria can lead to severe under- or over-selection and thus poor performance in high-dimensional settings (Liu *et al.* 2010).

### 2.3 The stabJGL algorithm

**Figure 1.**
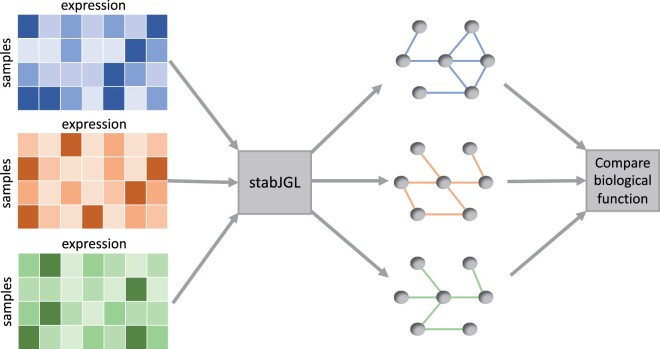
The workflow of stabJGL, where the network structures of different data types or conditions are jointly estimated and can then be compared.

To improve the performance of the JGL with the fused penalty for omics applications and other high-dimensional problems, we propose the stabJGL algorithm for stable sparsity and similarity selection in multiple-network reconstruction. StabJGL jointly estimates multiple networks by leveraging their common information and gives a basis for deeper exploration of their differences, as shown in Fig. 1. Below we outline the algorithm, which comprised two steps: (i) selecting the sparsity parameter $\lambda_{1}$ in the fused penalty (2) based on the notion of model stability, and (ii) selecting the similarity parameter $\lambda_{2}$ based on model likelihood. The full StabJGL algorithm is given in Supplementary Algorithm S1.

#### 2.3.1 Selecting $\lambda_{1}$

We first select $\lambda_{1}$ by extending the framework introduced by Liu *et al.* (2010) in their Stability Approach to regularization Criterion (StARS) to a multiple-network setting. The aim is to select the least amount of penalization that makes graphs sparse as well as reproducible under random subsampling. This is done by drawing many random subsamples from each of the *K* data types and using them to construct JGL graphs over a range of $\lambda_{1}$ values. The smallest parameter value for which a given graph estimation variability measure does not surpass a specified threshold is then selected. We use a measure of edge assignment instability across subsamples to quantify the variability.

Specifically, we consider a grid of $\lambda_{1}$ values in a suitable interval, i.e. (0,1] and keep the similarity parameter $\lambda_{2}$ fixed to some small value, such as 0.01 in the first instance. For $\eta =1,\ldots ,N_{\text{sample}}$, we draw a random subsample from each group *k’*s set of $n_{k}$ observations without replacement, each of size $b_{k}<n_{k}$. For each value of $\lambda_{1}$ to consider, we next construct the corresponding set of JGL graphs ${\left\{G_{\left(k\right)}^{\eta }(\lambda_{1})\right\}}_{k=1}^{K}$ from these *K* sets of subsamples, using the fused penalty (2).

The following is then done for each value of $\lambda_{1}$ we consider. For each group k=1,…,K and all possible node pairs (i,j) we estimate the probability of an edge between the nodes over the $N_{\text{sample}}$ inferred sets of graphs

(3) $${\hat{\psi }}_{ij}^{\left(k\right)}(\lambda_{1})=\frac{1}{N_{\text{sample}}}\sum_{\eta =1}^{N_{\text{sample}}}1\left[\left(i,j)\in G_{\left(k\right)}^{\eta }(\lambda_{1}\right)\right],$$

where 1[⋅] is the indicator function. Using this estimated probability, we find

(4) $${\hat{\xi }}_{ij}^{\left(k\right)}(\lambda_{1})=2{\hat{\psi }}_{ij}^{\left(k\right)}(\lambda_{1})(1-{\hat{\psi }}_{ij}^{\left(k\right)}(\lambda_{1})),$$

which is an estimate of two times the variance of the Bernoulli indicator of the edge (i,j) in group *k*. It lies in [0, 0.5] and can be regarded as an estimate of the proportion of times two inferred graphs for group *k* found with the given $\lambda_{1}$ value will disagree on the presence of the edge (i,j). Due to the $L_{1}$ penalty in (2), the number of inferred edges will decrease as $\lambda_{1}$ is increased.

For a given $\lambda_{1}$, ${\hat{\xi }}_{ij}^{\left(k\right)}(\lambda_{1})$ can be regarded as a measure of the variability of the edge (i,j) in group *k* across subsamples, and the total variability of graph *k* can be measured by averaging over all edges, yielding the estimate

(5) $${\hat{D}}_{\left(k\right)}(\lambda_{1})=\frac{1}{\left(\begin{array}{c} p\\ 2 \end{array}\right)}\sum_{i<j}{\hat{\xi }}_{ij}^{\left(k\right)}(\lambda_{1}).$$

For each value of $\lambda_{1}$, the total variability of the whole set of graphs found by the JGL is then found by averaging the variability over all *K* networks

(6) $$\hat{D}(\lambda_{1})=\frac{1}{K}\sum_{k=1}^{K}{\hat{D}}_{\left(k\right)}(\lambda_{1}).$$

For sufficiently large $\lambda_{1}$, all edges are excluded from the model and so the variability $\hat{D}(\lambda_{1})$ will be 0. The variability will in general increase as the penalty $\lambda_{1}$ decreases, however, for small enough $\lambda_{1}$ the graphs will become so dense that the variability starts to decrease again. As sparse network inference is the aim, we therefore monotonize the variability function by letting $\bar{D}(\lambda_{1})=\underset{t\geq \lambda_{1}}{\sup}\hat{D}(t)$.

Finally, for a given variability threshold $\beta_{1}$, the optimal penalty is chosen to be ${\hat{\lambda }}_{1}=\inf\{\lambda_{1}:\bar{D}(\lambda_{1})\leq \beta_{1}\}$. As opposed to $\lambda_{1}$, $\beta_{1}$ is an interpretable quantity and we propose a default threshold of $\beta_{1}=0.1$ as suggested by Liu *et al.* for the original StARS algorithm, which reflects an acceptance of 10% variability in the edge assignments.

#### 2.3.2 Selecting $\lambda_{2}$

After $\lambda_{1}$ has been selected, we select $\lambda_{2}$ with a multiple-network version of the extended BIC (eBIC or ${\text{BIC}}_{\gamma }$) of Foygel and Drton (2010). The eBIC is an extension of the Bayesian Information Criterion of Schwarz (1978), where the prior is reformulated to account for high-dimensional graphical settings. We propose an adaptation the eBIC to a multiple-network setting,

(7) $$\begin{array}{c} {\text{BIC}}_{\gamma }(\lambda_{1},\lambda_{2})=\sum_{k=1}^{K}[n_{k}\text{tr}(S^{\left(k\right)}{\hat{\Theta }}_{\lambda_{1},\lambda_{2}}^{\left(k\right)})-n_{k}\;\text{log}(\det({\hat{\Theta }}_{\lambda_{1},\lambda_{2}}^{\left(k\right)}))\\ +|E_{k}|\;\text{log}\;n_{k}+4|E_{k}|\gamma \;\text{log}\;p], \end{array}$$

where ${\hat{\Theta }}_{\lambda_{1},\lambda_{2}}^{\left(k\right)}$ is the estimated precision matrix of network *k* obtained with the penalty parameters $\lambda_{1}$ and $\lambda_{2}$, γ∈[0,1] is an additional penalty parameter and $\left|E_{k}\right|$ is the size of the corresponding edge set. A grid of $\lambda_{2}$ values is considered, with $\lambda_{1}$ fixed to the value selected in the previous step. The value of $\lambda_{2}$ that minimizes (7) is selected, and the final graph estimate is obtained by running the JGL with the selected penalty parameters $\lambda_{1}$ and $\lambda_{2}$ in the fused penalty (2). An investigation into the effect of selecting the two penalty parameters in reverse order is detailed in Supplementary Section S5, where we find that selecting $\lambda_{1}$ after $\lambda_{2}$ might be desired in settings where one is not concerned about a high false discovery rate.

#### 2.3.3 Implementation details

StabJGL is implemented in R, and available as an R package at https://github.com/Camiling/stabJGL. The subsampling routine is implemented so it can be done in parallel. The JGL fittings are done as in Danaher *et al.* (2014), using an ADMM (Alternating Direction Method of Multipliers) algorithm (Boyd *et al.* 2010) for general penalty functions to solve the penalized log-likelihood problem (1) with the fused penalty (2). The stabJGL algorithm itself does not inherently handle missing data, and requires complete data for the precision matrix estimation. In the case of missing data, we recommend using state-of-the-art approaches, such as multiple imputation to defer the issue. By default, 20 subsamples are used and we evaluate 20 values each of $\lambda_{1}\in [0.01,1]$ and $\lambda_{2}\in [0,0.1]$. We use a subsample size of $b_{k}=\left\lfloor 10\sqrt{n_{k}}\right\rfloor$ for group k=1,…,K, which as Liu *et al.* (2010) show in a single network setting maintains theoretical properties for containing the true graph with high probability as well as high empirical performance. The additional penalty parameter γ in the eBIC for similarity selection is set to 0 by default, corresponding to the standard BIC. The choice of γ is not crucial as we are using it for similarity and not sparsity selection, and thus compare graphs of similar sparsity. We found this value to be suitable in most applications but leave the option to increase the penalization. We employ a default variability threshold of $\beta_{1}=0.1$.

## 3 Results

### 3.1 Simulated data

We first assess the performance of stabJGL on simulated data. We compare the network reconstruction ability of stabJGL to that of state-of-the-art methods, including the JGL with the fused penalty (FGL) and group penalty (GGL) with penalty parameters selected with the default AIC-based criterion (Danaher *et al.* 2014). To assess the performance of another selection criterion specifically designed for high-dimensional graph selection, we also consider FGL with penalty parameters tuned by the extended BIC for multiple graphs (7). We further include the Bayesian spike-and-slab JGL (SSJGL) of Li *et al.* (2019b), as well as the Glasso of Friedman *et al.* (2008) tuned by StARS (Liu *et al.* 2010). The latter estimates each network separately. We generate data that closely resembles our omics application of interest, featuring partial correlations between 0.1 and 0.2 in absolute value, while also exhibiting the “scale-free” property—a typical assumption for omics data where the “degree distribution” (i.e. the distribution of the number of edges that are connected to the nodes) adheres to a power-law distribution (Chen and Sharp 2004). We consider a wide range of settings, simulating K∈{2,3,4} networks with p∈{100,200,300,1000} nodes. We manipulate the degree of similarity in their “true” graphical structures to assess the performance of the method over a wide range of scenarios. We then apply different network reconstruction techniques to determine the networks from the data and report the results averaged over N=100 replicates. The parameter specifications for the different methods are given in Supplementary Section S2. We also investigate the effect of the variability threshold $\beta_{1}$ in stabJGL on the results in a setting with p=100 nodes and K=2 networks. Finally, to compare the scalability of the respective methods, we consider the time needed to infer networks for various *p* and *K*. Further details and code for the simulation study can be found at https://github.com/Camiling/stabJGL_simulations.

Estimation accuracy is assessed with the “precision” (positive predictive value), and the “recall” (sensitivity). The precision gives the fraction of predicted edges that were correct, while the recall is the fraction of edges in the true graph that were identified by the inference. Because the sparsity of estimated networks will vary between methods, the precision–recall trade-off should be taken into consideration. In general, the recall will increase with the number of selected edges while the precision will decrease. Since sparsity selection is a main feature of our proposed method, we do not consider threshold-free comparison metrics, such as the AUC. We therefore put emphasis on the following characteristics in our comparative simulation study; (i) suitable sparsity level selection, (ii) utilization of common information at any level of network similarity, i.e. inference improves with increased network similarity, and (iii) a suitable precision–recall trade-off that overly favors either measure.

### 3.2 Simulation results

**Figure 2.**
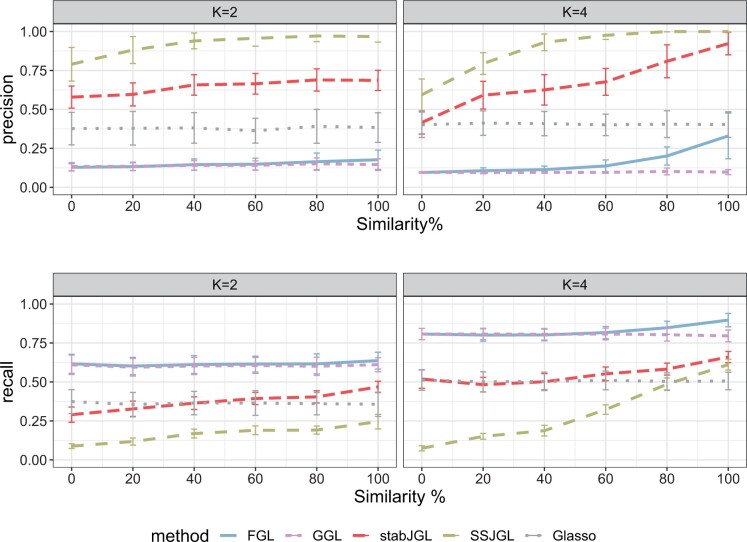
Performance of the Glasso, FGL, and GGL tuned by AIC, SSJGL, and stabJGL, reconstructing K∈{2,4} graphs with p=100 nodes and various similarity of the true graph structures. The similarity between the graphs is shown as the percentage of edges they have in common. The results are averaged over N=100 replicates and show the precision and recall for the first estimated graph in each setting, reconstructed from n∈{100,150} observations and n∈{150,200,250,300} observations for K=2 and K=4, respectively. Standard deviation bars are shown for all methods. All graphs have true sparsity 0.02.

The results for K=2 and K=4 networks with p=100 nodes are summarized in Fig. 2. The full tables of results for these settings and the additional settings with K∈{2,3,4} networks and p∈{100,200,300,1000} nodes are shown in Supplementary Section S2, where details on selected sparsity and penalty parameters are given for all methods. Additional simulations investigating the effect of random noise on the graph reconstruction performance of the different methods are also given in Supplementary Section S6. In Fig. 2, FGL tuned with eBIC did not select any edges in any of the settings and is therefore not shown. In all settings considered, the FGL and GGL with the default AIC-based penalty parameter selection strongly over-select edges. This leads to high recall, but very low precision. Second, they do not appear to sufficiently utilize network similarities; the performance of the two methods, particularly GGL, differs little between completely unrelated and identical networks. Notably, in all cases, the selected value of $\lambda_{2}$ is smaller for FGL and GGL tuned by AIC than it is for stabJGL. Consequently, similarity is not sufficiently encouraged even in settings where the networks are identical. The AIC criterion does not seem to provide sufficient penalization to encourage suitable sparsity and similarity. On the other hand, the alternative eBIC criterion gives extremely sparse FGL estimates, selecting an empty graph in most settings, i.e. no edges. Although the extended BIC is developed specifically for graphical model selection, likelihood-based criteria for sparsity selection tend to perform poorly in high-dimensional settings and risk both severe under- and over-selection (Foygel and Drton 2010). This issue is avoided in the stabJGL algorithm as the eBIC only is used to select similarity and not sparsity.

The Bayesian SSJGL tends to select very few edges, leading to high precision but low recall. Its performance deteriorates drastically as the network differences increase, leading to extremely low recall. This implies a lack of flexibility to adapt to varying network similarity levels, as has previously been observed (Lingjærde *et al.* 2022). Out of all the joint methods, stabJGL gives the most accurate sparsity estimate. This ensures that we neither get very low precision like FGL and GGL tuned by AIC, nor very low recall like SSJGL and FGL tuned by eBIC. Replacing the mean of the $\lambda_{1}$’s over the N=100 replicates by alternative measures, such as the median yields the same reported penalty parameter values and hence results in all settings considered in Fig. 2. The mean and median could be expected to differ slightly if a finer grid of $\lambda_{1}$ values were to be considered, but our findings suggest they would yield highly similar results. StabJGL also appears to adapt well to the similarity between networks, with the prediction accuracy increasing with the number of shared edges. As a result, the method either outperforms the Glasso tuned by StARS for highly similar networks or performs comparably to it for unrelated networks. The similar performance for unrelated networks can be explained by the fact that the sparsity controlling penalty parameters of both methods are tuned with a stability-based approach. The results suggest that stabJGL adapts well to the level of similarity and hence can be employed agnostically in settings where there is no prior knowledge about the degree to which the networks have shared structures.

A key question is whether stabJGL can achieve as high precision as the methods that give sparser networks (i.e. SSJGL) by using a lower variability threshold. Similarly, we want to see if stabJGL can achieve as high recall as the methods that infer more edges (i.e. FGL and GGL). To investigate this, we consider the same setting as in Fig. 2 with K=2 networks. Supplementary Figure S4 compares the performance of stabJGL for different values of the variability threshold $\beta_{1}$ to the other methods, and we find that by decreasing (resp. increasing) $\beta_{1}$ we can obtain at least as high precision (resp. recall) as the other methods at any level of similarity. This illustrates that the method can be adapted to reflect the priorities of the user (i.e. concern for false positives versus false negatives). For most applications, a middle-ground value, such as 0.1 yields a good balance between false positives and false negatives as demonstrated in the simulations.

### 3.3 Runtime profiling

**Figure 3.**
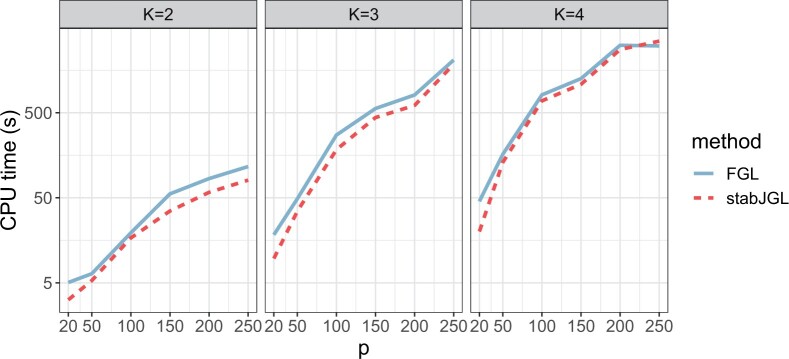
CPU time in seconds on a logarithmic scale used to jointly infer networks for K∈{2,3,4} networks and various numbers of nodes *p*, with n∈{100,150} observations, for FGL tuned with AIC and stabJGL. The computations were performed on a 16-core Intel Xeon CPU, 2.60 GHz.

Figure 3 shows the CPU time used to jointly infer networks for K∈{2,3,4} networks and various numbers of nodes *p*, with n∈{100,150} observations, for the JGL with the fused penalty (FGL) with penalty parameters tuned with the AIC and stabJGL with the same parameter specifications as in the previously described simulations. Due to an efficient parallelized implementation, stabJGL has an almost identical runtime to FGL when the same number of $\lambda_{1}$ and $\lambda_{2}$ values are considered. Thus, the increased estimation accuracy of stabJGL does not come at a computational cost. It is important to note that due to the generalized fused lasso problem having a closed-form expression for the updates in the special case of K=2 (Danaher *et al.* 2014), stabJGL is substantially faster for only two networks than for K>2. As stabJGL uses the fused penalty this comparison is the most relevant, but a runtime comparison of all methods considered in our simulation study can be found in Supplementary Fig. S1. In the Supplementary Material, we also demonstrate that stabJGL can be applied to problems with p≥2500 nodes or K=10 networks within reasonable time (Supplementary Fig. S3).

### 3.4 Pan-cancer data

We perform a proteomic network analysis of Reverse Phase Protein Array (RPPA) data from The Cancer Genome Atlas (TCGA) across different pan-Cancer tumor types (Cancer Genome Atlas Network and others 2012). In a large proteomic pan-Cancer study of 11 TCGA tumor types, Akbani *et al.* (2014) identified a major tumor super cluster consisting of hormonally responsive “women’s cancers” [Luminal breast cancer (BRCA), ovarian cystadenocarcinoma (OVCA), and uterine corpus endometrial carcinoma (UCEC)]. Our objective is to map the proteomic network structure of the respective tumor types, so that we can get a better grasp of the common mechanisms at play in the hormonally responsive tumors. We are also interested in highlighting the differences.

We consider RPPA data from Luminal BRCA (n=273), high-grade serous OVCA (n=412), and UCEC (n=404). All data are downloaded from the UCSC Xena Browser (Goldman *et al.* 2020). The data are measured with p=131 high-quality antibodies that target (phospho)-proteins. To alleviate batch effects, the RPPA data are normalized with replicate-base normalization (Akbani *et al.* 2014). We use stabJGL to jointly estimate the proteomic networks of the respective tumor types and interpret the results and their implications. We compare the output with that obtained with the FGL of Danaher *et al.* (2014) with the default penalty parameter tuning with AIC as described in Section 3.1. Further details and code for the analysis are given at https://github.com/Camiling/stabJGL_analysis.

### 3.5 Pan-cancer analysis results

#### 3.5.1 Estimated proteomic networks

**Figure 4.**
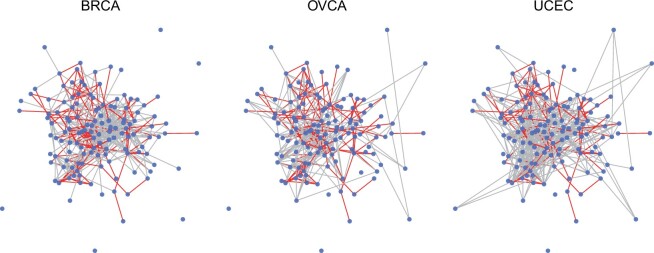
Proteomic network structure identified by stabJGL for the BRCA, OVCA, and UCEC tumors. The nodes represent proteins, and edges common to all three networks are darker.

The resulting stabJGL proteomic networks of the three tumor types are shown in Fig. 4, where we observe plenty of common edges as well as network-specific ones. The sparsity as well as the selected penalty parameter values in the resulting stabJGL and FGL networks is shown in Table 1. The tendency as observed in the simulations of FGL tuned by the AIC to over-select edges appears to be consistent with the findings in this context. With more than two-thirds of all potential edges being determined as present by FGL, the results are challenging to interpret and derive meaningful conclusions from. From a biological standpoint, we would not expect a proteomic network to be this saturated in terms of associations due to the expected scale-free property of the degree distribution (Barabasi and Oltvai 2004). While the degree distributions of the sparse stabJGL networks all follow a power-law with many low-degree nodes and fewer high-degree ones (hubs), an expected trait for omics data (Chen and Sharp 2004), the degree distributions of the FGL networks do not. The full degree distributions are shown in Supplementary Fig. S6.

**Table 1.** Network analysis results for stabJGL and FGL tuned by the AIC, applied to data from BRCA, OVCA, and UCEC tumors.

			Sparsity		
	$\lambda_{1}$	$\lambda_{2}$	BRCA	UCEC	OVCA
FGL	0.010	0.000	0.689	0.709	0.679
stabJGL	0.323	0.008	0.049	0.036	0.039

In terms of penalty parameters, we see that just like for the simulated data the AIC selects very small penalty parameters for FGL, resulting in little sparsity and similarity encouragement. Given the findings of Akbani *et al.* (2014) about the presence of a super cluster consisting of the three hormonally responsive cancer types, it is not unreasonable to expect at least some proteomic network similarity to be encouraged by a joint method. This is achieved by stabJGL, which selects a large enough value of $\lambda_{2}$ to encourage similarity. A comparison of the pairwise similarities of the proteomic networks reveals that stabJGL indeed finds the networks of the three tumor types to be more similar than FGL, in accordance with the findings of Akbani *et al.* (2014) (Supplementary Fig. S5).

#### 3.5.2 Edge validation in STRING

To compare the level of evidence supporting the edges detected by stabJGL and FGL tuned by the AIC in the literature, we conduct edge validation using the STRING database of known and predicted protein–protein interactions (Szklarczyk *et al.* 2019). To ensure the reliability of the validation process, we only consider the experimentally validated interactions in STRING as evidence, with default confidence score threshold ≥0.4. The number of predicted edges with supporting evidence in the STRING database, as well as the proportion of all predicted edges that have evidence in STRING, is computed for the respective stabJGL and FGL networks and shown in Table 2. While the fused Glasso identifies a larger number of edges with evidence in STRING, the method also includes far more edges than stabJGL, resulting in an overall lower percentage of edges with supporting evidence in the STRING database than stabJGL, for all three tumor types investigated. Complete lists of the protein–protein edges identified by stabJGL for each tumor type are provided as Supplementary Files.

**Table 2.** Comparison of evidence for edges in the respective FGL tuned by AIC and stabJGL proteomic networks of BRCA, OVCA, and UCEC tumors, considering experimentally determined protein–protein interactions documented in the STRING database.^a^

	Edge evidence %		Edge evidence #	
Dataset	FGL (%)	stabJGL (%)	FGL	stabJGL
BRCA	5.4	**12.3**	408	68
UVEC	5.6	**10.0**	398	44
OVCA	5.7	**12.4**	424	42

a Both the percentage of predicted edges with evidence and the number of predicted edges with evidence are shown. The highest percentage of edges with evidence is in bold.

#### 3.5.3 Findings consistent with literature

StabJGL successfully identifies protein–protein interactions known from literature. To highlight the findings of the proposed methodology, we only discuss edges and central proteins identified by stabJGL but not FGL. One example is the edge between activated (S345-phosphorylated) Checkpoint kinase 1 (Chk1) and DNA repair protein RAD51 homolog 1 (Rad51) in ovarian and BRCA. The complex between the tumor suppressor BRCA2, which manifests predominantly in ovarian and BRCA, and Rad51, is mediated by the DNA damage checkpoint Chk1 through Rad51 phosphorylation (Bahassi *et al.* 2008, Nair *et al.* 2020). It is also reassuring that stabJGL identifies many relevant tumor type-specific proteins as hubs in the relevant tumor type only, such as mammalian target of rapamycin (mTOR), Tuberous Sclerosis Complex 2, and Ribosomal protein S6 in BRCA, all of which are involved or up/downstream of the PI3K/AKT/mTOR pathway known to frequently be deregulated in Luminal BRCA (Miricescu *et al.* 2020). Lists of the top hubs in the respective stabJGL and FGL networks of the different tumor types, and their node degree, are given in Supplementary Tables S9 and S10.

**Figure 5.**
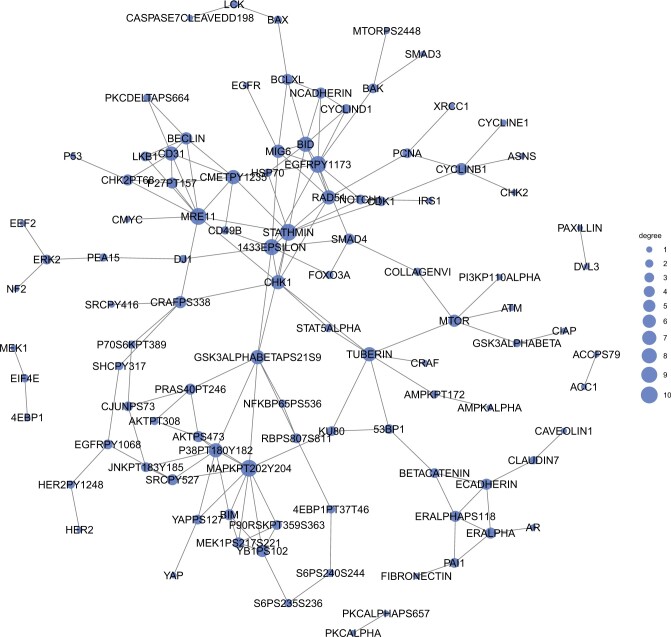
The proteomic network structure identified by stabJGL common to all three tumor types (BRCA, UCEC, and OCVA). The node size indicates the degree in the common network structure, with proteins with more edges being represented by larger nodes.

StabJGL also captures edges that we expect to be present in all three tumor types, such as the known interaction between the transcription factor Forkhead box O3 and 14–3-3-epsilon, which facilitates cancer cell proliferation (Nielsen *et al.* 2008, Tzivion *et al.* 2011). This common interaction is documented in the STRING database. Figure 5 shows the network structure identified by stabJGL that is common to all three tumor types. Central proteins in this common network structure include Oncoprotein 18 (Stathmin), which is known to be relevant in all three hormonally responsive cancers due to its role in the regulation of cell growth and motility (Bieche *et al.* 1998, Belletti and Baldassarre 2011, Trovik *et al.* 2011).

#### 3.5.4 Potential candidate hubs

The recovery of documented links in the protein networks estimated by stabJGL highlights its capability to detect numerous relevant proteins and interactions. The potential for new discoveries is however an important aspect of stabJGL, as suggested by its good performance on simulated data. For example, stabJGL identifies phosphorylated epidermal growth factor receptor (EGFR) as a central hub protein in all three tumor types. While known to be relevant in ovarian cancer (Yang *et al.* 2013, Zhang *et al.* 2016), the role of activated EGFR in UCEC and Luminal BRCA and is not yet clarified. Our findings suggest it could be relevant in all three hormonally responsive tumor types. Further, Platelet endothelial cell adhesion molecule (CD31) is found to be a central protein in UCEC only. The protein is important for angiogenesis and has been implicated in other tumor types, such as hemangioma (Bergom *et al.* 2005). Its prominence in the proteomic UCEC network suggests it may play a crucial role in this tumor type as well. Overall, these results showcase how stabJGL can aid in generating hypotheses by identifying central proteins and associations.

## 4 Discussion

Suitable sparsity and similarity selection is key for capturing and studying multiple-related biological networks. We have proposed the stabJGL algorithm, which determines the penalty parameters in the fused Glasso for multiple networks based on the principle of model stability. StabJGL demonstrably avoids the under- or over-selection of edges observed in state-of-the-art selection methods based on information criteria and succeeds at leveraging network similarities to a suitable degree. Consequently, the method can be employed in situations where the actual degree of similarity is uncertain, resulting in marked benefits with minimal risks associated with its use. StabJGL offers a fast parallelized implementation, particularly for K=2 networks as a closed-form expression of the updates exists. We successfully apply the method to problems with p>2000 nodes or K>10 networks.

With our novel approach, we can identify both common and distinct mechanisms in the proteomic networks of different types of hormonally responsive women’s cancers. The results obtained with stabJGL are in line with known biology and compliment those of Akbani *et al.* (2014) by offering additional understanding of the underlying mechanisms in action. By recognizing various proteins as highly critical in the proteomic networks, stabJGL suggests their possible involvement in driving the diseases. The method both identifies proteins that are central in all three hormonally responsive cancers (e.g. phosphorylated EGFR) and proteins of tumor-specific relevance (e.g. CD31 in UCEC).

The proposed approach can be applied to any type of omics data that can be transformed to adhere to the normality assumption. It is however important to note that if the datasets that are to be jointly analyzed are different types of omics data, a one-to-one mapping between the nodes in each dataset would be needed, e.g. having protein and mRNA expression data corresponding to the “same” encoding genes.

In the setting of the data sources being from the same set of patients, the joint approach is not applicable due to the key assumption that all observations are independent. One such example could be when the data sources are mRNA and protein expression from the same patients. In that case, alternative approaches developed for multi-omic data integration might be more suitable, see, e.g. Bonnet *et al.* (2015) and Li and Jackson (2015). In the case of multiple shared axes, an extension of our approach could be to concatenate the different omics data types and determine the global network. If independent observations from multiple sources, such as clinical groups or conditions is available as well, stabJGL could then be used to determine links across and within omics data types simultaneously. Such an approach might warrant the introduction of different penalties in (1), both within and across the different omics data types.

While stabJGL is highly competitive in terms of scalability relative to the existing methodology, all algorithms for estimating Gaussian graphical models have inevitable matrix inversion steps. This is computationally demanding for large *p*, and, at present, joint graphical methods based on Gaussian graphical models are realistically not feasible for the analysis of extremely high-dimensional data with tens of thousands of nodes. It can be argued that even if such an analysis was possible, it is not sensible to attempt to determine the conditional independence structure for problems of this size. For example, if we were to consider the expression of p=20 000 genes and K=3 groups, we would have the problem of determining over half a billion edges. With only a couple hundred samples in most studies and hence little statistical power, low estimation accuracy and many false discoveries would be expected. Meaningful interpretation of the results would also be a challenge. In settings where *p* is extremely large, an option could instead be to use a fast marginal correlation-based network approach, such as the weighted gene co-expression network analysis method of Langfelder and Horvath (2008), to identify subnetworks that are relevant for a more refined conditional independence network analysis.

Future extensions of the method can include alternative measures of variability, such as the entropy [see, e.g. Lartigue *et al.* (2020)]. Further, while the method is formulated specifically for the JGL with the fused penalty, it can in principle be used for any joint network approach requiring the tuning of sparsity- and similarity controlling parameters. Another relevant extension could be the introduction of network-specific sparsity penalty parameters $\lambda_{1,k}$ and network pair-specific similarity penalty parameters $\lambda_{2,kl}$ for graphs 1≤k<l≤K, which would provide additional flexibility in terms of differing levels of sparsity and similarity across networks. The introduction of these parameters would however invoke a sharp increase in computational complexity, proportional to $K^{2}$.

To conclude, stabJGL provides a reliable approach to joint network inference of omics data. The output can provide a better understanding of both common and data type-specific mechanisms, which can be used for hypothesis generation regarding potential therapeutic targets.

## Supplementary Material

vbad185_Supplementary_Data

## Data Availability

The TCGA data sets analysed in this article is publicly available in the University of California Santa Cruz Xena Browser repository (http://xena.ucsc.edu), from where we downloaded RPPA data for BRCA (dataset ID: TCGA.BRCA.sampleMap/RPPA_RBN), UCEC (dataset ID: TCGA.UCEC.sampleMap/RPPA_RBN) and OVCA (dataset ID: TCGA.OV.sampleMap/RPPA_RBN).

## References

[vbad185-B1] Akaike H , Petrov BN, Csaki F. Information theory and an extension of the maximum likelihood principle. In: *Second International Symposium on Information Theory*, New York, NY. New York: Springer, 1973, 267–81.

[vbad185-B2] Akbani R , Ng PKS, Werner HM et al A pan-cancer proteomic perspective on The Cancer Genome Atlas. Nat Commun 2014;5:3887. 24871328 10.1038/ncomms4887 PMC4109726

[vbad185-B3] Bahassi E , Ovesen J, Riesenberg A et al The checkpoint kinases Chk1 and Chk2 regulate the functional associations between hBRCA2 and Rad51 in response to DNA damage. Oncogene 2008;27:3977–85. 18317453 10.1038/onc.2008.17

[vbad185-B4] Barabasi A-L , Oltvai ZN. Network biology: understanding the cell’s functional organization. Nat Rev Genet 2004;5:101–13. 14735121 10.1038/nrg1272

[vbad185-B5] Belletti B , Baldassarre G. Stathmin: a protein with many tasks. New biomarker and potential target in cancer. Expert Opin Ther Targets 2011;15:1249–66. 21978024 10.1517/14728222.2011.620951

[vbad185-B6] Bergom C , Gao C, Newman PJ. Mechanisms of PECAM-1-mediated cytoprotection and implications for cancer cell survival. Leuk Lymphoma 2005;46:1409–21. 16194886 10.1080/10428190500126091

[vbad185-B7] Bieche I , Lachkar S, Becette V et al Overexpression of the stathmin gene in a subset of human breast cancer. Br J Cancer 1998;78:701–9. 9743287 10.1038/bjc.1998.565 PMC2062973

[vbad185-B8] Bonnet E , Calzone L, Michoel T. Integrative multi-omics module network inference with lemon-tree. PLoS Comput Biol 2015;11:e1003983. 25679508 10.1371/journal.pcbi.1003983 PMC4332478

[vbad185-B9] Boyd S , Parikh N, Chu E et al Distributed optimization and statistical learning via the alternating direction method of multipliers. FNT Mach Learn 2010;3:1–122.

[vbad185-B10] Cancer Genome Atlas Network and others. Comprehensive molecular portraits of human breast tumours. Nature 2012;490:61–70. 23000897 10.1038/nature11412 PMC3465532

[vbad185-B11] Chen H , Sharp BM. Content-rich biological network constructed by mining PubMed abstracts. BMC Bioinformatics 2004;5:147. 15473905 10.1186/1471-2105-5-147 PMC528731

[vbad185-B12] Danaher P , Wang P, Witten DM. The joint graphical lasso for inverse covariance estimation across multiple classes. J R Stat Soc Series B Stat Methodol 2014;76:373–97. 24817823 10.1111/rssb.12033 PMC4012833

[vbad185-B13] Fan J , Feng Y, Wu Y. Network exploration via the adaptive LASSO and SCAD penalties. Ann Appl Stat 2009;3:521. 21643444 10.1214/08-AOAS215SUPP PMC3105782

[vbad185-B14] Foygel R , Drton M. Extended Bayesian information criteria for Gaussian graphical models. In *Advances in Neural Information Processing Systems, Vancouver, BC*, 604–12. San Diego, CA: NeurIPS, 2010.

[vbad185-B15] Friedman J , Hastie T, Tibshirani R. Sparse inverse covariance estimation with the graphical lasso. Biostatistics 2008;9:432–41. 18079126 10.1093/biostatistics/kxm045 PMC3019769

[vbad185-B16] Goldman MJ , Craft B, Hastie M et al Visualizing and interpreting cancer genomics data via the Xena platform. Nat Biotechnol 2020;38:675–8. doi: 10.1038/s41587-020-0546-8 32444850 PMC7386072

[vbad185-B17] Guo J , Levina E, Michailidis G et al Joint estimation of multiple graphical models. Biometrika 2011;98:1–15. 23049124 10.1093/biomet/asq060 PMC3412604

[vbad185-B18] Langfelder P , Horvath S. WGCNA: an R package for weighted correlation network analysis. BMC Bioinformatics 2008;1:559. 10.1186/1471-2105-9-559 PMC2631488 19114008

[vbad185-B19] Lartigue T , Bottani S, Baron S et al Gaussian graphical model exploration and selection in high dimension low sample size setting. IEEE Trans Pattern Anal Mach Intell 2020;43:3196–213. 10.1109/TPAMI.2020.2980542 32175856

[vbad185-B20] Lauritzen SL. Graphical Models. Vol. 17. Oxford, UK: Clarendon Press, 1996.

[vbad185-B21] Li Y , Craig BA, Bhadra A. The graphical horseshoe estimator for inverse covariance matrices. J Comput Graph Stat2019a;28:747–57.

[vbad185-B22] Li Y , Jackson SA. Gene network reconstruction by integration of prior biological knowledge. G3 (Bethesda) 2015;5:1075–9. doi: 10.1534/g3.115.018127. 25823587 PMC4478538

[vbad185-B23] Li Z , Mccormick T, Clark S. Bayesian joint spike-and-slab graphical lasso. In: *International Conference on Machine Learning, Long Beach, CA*. 3877–85. PMLR, 2019b. PMC7845917 33521648

[vbad185-B24] Lingjærde C , Fairfax BP, Richardson S et al Scalable multiple network inference with the joint graphical horseshoe. Ann Appl Stat 2023; in press.

[vbad185-B25] Lingjærde C , Lien TG, Borgan Ø et al Tailored graphical lasso for data integration in gene network reconstruction. BMC Bioinformatics 2021;22:498. 34654363 10.1186/s12859-021-04413-z PMC8518261

[vbad185-B26] Liu H , Roeder K, Wasserman L. Stability approach to regularization selection (StARS) for high dimensional graphical models. Adv Neural Inf Process Syst 2010;24:1432–40. 25152607 PMC4138724

[vbad185-B27] Meinshausen N , Bühlmann P. High-dimensional graphs and variable selection with the lasso. Ann Statist 2006;34:1436–62.

[vbad185-B28] Miricescu D , Totan A, Stanescu-Spinu I-I et al PI3K/AKT/mTOR signaling pathway in breast cancer: from molecular landscape to clinical aspects. Int J Mol Sci 2020;22:173. 33375317 10.3390/ijms22010173 PMC7796017

[vbad185-B29] Nair J , Huang T-T, Murai J et al Resistance to the CHK1 inhibitor prexasertib involves functionally distinct CHK1 activities in BRCA wild-type ovarian cancer. Oncogene 2020;39:5520–35. 32647134 10.1038/s41388-020-1383-4 PMC7426265

[vbad185-B30] Nielsen MD , Luo X, Biteau B et al 14-3-3-Epsilon antagonizes FoxO to control growth, apoptosis and longevity in Drosophila. Aging Cell 2008;7:688–99. 18665908 10.1111/j.1474-9726.2008.00420.x PMC3851013

[vbad185-B31] Peterson C , Stingo FC, Vannucci M. Bayesian inference of multiple Gaussian graphical models. J Am Stat Assoc 2015;110:159–74. 26078481 10.1080/01621459.2014.896806 PMC4465207

[vbad185-B32] Schwarz G. Estimating the dimension of a model. Ann Statist 1978;6:461–4.

[vbad185-B33] Szklarczyk D , Gable AL, Lyon D et al STRING v11: protein-protein association networks with increased coverage, supporting functional discovery in genome-wide experimental datasets. Nucleic Acids Res 2019;47:D607–13. 30476243 10.1093/nar/gky1131 PMC6323986

[vbad185-B34] Trovik J , Wik E, Stefansson IM et al; MoMaTec Study Group. Stathmin overexpression identifies high-risk patients and lymph node metastasis in endometrial cancer. Clin Cancer Res 2011;17:3368–77. 21242118 10.1158/1078-0432.CCR-10-2412

[vbad185-B35] Tzivion G , Dobson M, Ramakrishnan G. FoxO transcription factors; Regulation by AKT and 14-3-3 proteins. Biochim Biophys Acta 2011;1813:1938–45. 21708191 10.1016/j.bbamcr.2011.06.002

[vbad185-B36] Wang H. Bayesian graphical lasso models and efficient posterior computation. Bayesian Anal 2012;7:867–86.

[vbad185-B37] Wang H. Scaling it up: stochastic search structure learning in graphical models. Bayesian Anal 2015;10:351–77.

[vbad185-B38] Yang J-Y , Yoshihara K, Tanaka K et al; Cancer Genome Atlas (TCGA) Research Network. Predicting time to ovarian carcinoma recurrence using protein markers. J Clin Investig 2013;123:3740–50. 23945238 10.1172/JCI68509 PMC3754259

[vbad185-B39] Zhang H , Liu T, Zhang Z et al; CPTAC Investigators. Integrated proteogenomic characterization of human high-grade serous ovarian cancer. Cell 2016;166:755–65. 27372738 10.1016/j.cell.2016.05.069 PMC4967013

