# Supplementary material for: StabJGL: a stability approach to sparsity and similarity selection in multiple-network reconstruction
Source: Bioinform Adv. 2023 Dec 19;3(1):vbad185. doi: 10.1093/bioadv/vbad185 (PMC10751232; doi:10.1093/bioadv/vbad185)
Supplement: vbad185_Supplementary_Data [file vbad185_supplementary_data.zip › Supplement.pdf]

Supplement to “StabJGL: a stability approach to sparsity and similarity selection in multiple network reconstruction”

Camilla Lingjærde and Sylvia Richardson

S1 Algorithm

The full stabJGL algorithm is given in Algorithm S1. JGL($\cdot$) indicates that the joint graphical lasso function with the fused penalty is applied. The output of the JGL function can either be a set of graphs, a set of precision matrices or an edge set, depending on what is required Algorithm S1.

S2 Full simulation study

S2.1 Simulation study details

For FGL and GGL, the two penalty parameters are chosen in a sequential fashion with the default AIC-based criterion proposed by Danaher et al., 2014, with 20 values of $\lambda_1 \in [0.01,1]$ and $\lambda_2 \in [0,0.1]$ respectively being evaluated. We consider the eBIC criterion on the same grid of values for FGL, with a moderate value of $\gamma = 0.2$. We consider the same set of λ_1 and λ_2 values in the stabJGL algorithm and let $\gamma = 0$ in the eBIC criterion for similarity selection. For stabJGL and the graphical lasso tuned by StARS, we use a variability threshold of 0.1 and use 20 subsamples. For the Bayesian spike-and-slab joint graphical lasso all parameter specifications are as suggested by Li et al., 2019.

S2.2 Results

Simulation studies are conducted to assess a wide range of scenarios and compare the performance of the graphical lasso (Glasso), the fused joint graphical lasso (FGL) and the group joint graphical lasso (GGL) tuned by AIC, the fused joint graphical lasso tuned by eBIC, the Bayesian spike-and-slab joint graphical lasso (SSJGL) and stabJGL. Table S1 shows the network reconstruction performance of the methods in a $K = 3$ network setting with $p = 100$ nodes and various similarity of the true graph structures, averaged over $N = 100$ simulations. Similarly, Table S2 shows the network reconstruction performance of the methods in a $K = 3$ network setting with $p = 200$ nodes and various similarity of the true graph structures, averaged over $N = 100$ simulations. Similarly, Table S3 shows the results for a $K = 4$ setting with $p = 100$ nodes. In Table S4, the results are shown for a $K = 2$ network setting with $p = 100$ nodes. Finally, the results from a $K = 2$ network setting with $p = 300$ nodes are shown in Table S5 and the results from a $K = 2$ network setting with $p = 1000$ nodes are shown in Table S6. In the latter cases, due to the longer run time of SSJGL as demonstrated in Section S3, this method is omitted to make the simulation study feasible within reasonable time ($<$ 48 hours). We also only consider $N = 10$ simulations for $p = 1000$ nodes. The results from the additional simulation are in line with those from the main simulation study; stabJGL succeeds at capturing both the sparsity level and similarity between the networks to a better degree than FGL and GGl, while either outperforming the standard graphical lasso for highly similar networks or getting comparable results for unrelated networks. FGL with the alternative eBIC selection mostly selects empty graphs. Finally, SSJGL select very few edges, leading to high precision but very low recall in all cases.

S3 Runtime profiling

Figure S1 compares the time used to infer $K \in \{2, 4\}$ networks with various numbers of nodes p, for the different network reconstruction methods. We only consider the AIC selection for FGL as the eBIC considers the same grid of values and hence has identical running time. All methods are run with the same parameter specifications as in the main simulation study. The simulated networks are set to have

Algorithm S1 The stabJGL algorithm

Require: $n_k \times p$ data matrix $\boldsymbol{X}^{(k)}$ for $k = 1,\ldots,K$

$\Lambda_1 \leftarrow \{0.01,0.02,\ldots,1\}$, $\Lambda_2 \leftarrow \{0,0.01,\ldots,0.1\}$
$\lambda_2^{(\text{init})} \leftarrow 0.01$
$\beta_1 \leftarrow 0.1$
$N_{\text{sample}} \leftarrow 20$
$\gamma \leftarrow 0$
$b_k \leftarrow \lfloor 10\sqrt{n_k} \rfloor$ for $k = 1,\ldots,K$
$\boldsymbol{S}^{(k)} \leftarrow \frac{1}{n_k-1} {\boldsymbol{X}^{(k)}}^T \boldsymbol{X}^{(k)}$ for $k = 1,\ldots,K$
for λ_1 in Λ_1 **do**
    for $\eta = 1$ to N_{sample} **do**
        for $k = 1$ to K **do**
            Sample b_k indices $I_k \subset \{1,\ldots,n_k\}$
            $\boldsymbol{X}^{(k)}_{\text{sample}} \leftarrow \boldsymbol{X}^{(k)}[I_k,]$
        end for
        $\{G^{\eta}_{(k)}(\lambda_1)\}_{k=1}^K \leftarrow \text{JGL}\left(\{\boldsymbol{X}^{(k)}_{\text{sample}}\}_{k=1}^K \mid \lambda_1, \lambda_2^{(\text{init})}\right)$
    end for
    for $k = 1$ to K **do**
        for $j = 1$ to p **do**
            for $i = 1$ to $j-1$ **do**
                $\widehat{\psi}^{(k)}_{ij}(\lambda_1) \leftarrow \frac{1}{N_{\text{sample}}} \sum_{\eta=1}^{N_{\text{sample}}} \mathbb{1}\left[(i,j) \in G^{\eta}_{(k)}(\lambda_1)\right]$
                $\widehat{\xi}^{(k)}_{ij}(\lambda_1) \leftarrow 2\widehat{\psi}^{(k)}_{ij}(\lambda_1)(1 - \widehat{\psi}^{(k)}_{ij}(\lambda_1))$
            end for
        end for
        $\widehat{D}_{(k)}(\lambda_1) \leftarrow \frac{1}{\binom{p}{2}} \sum_{i<j} \widehat{\xi}^{(k)}_{ij}(\lambda_1)$
    end for
    $\widehat{D}(\lambda_1) \leftarrow \frac{1}{K} \sum_{k=1}^K \widehat{D}_{(k)}(\lambda_1)$
    $\bar{D}(\lambda_1) \leftarrow \sup_{t \geq \lambda_1} \widehat{D}(t)$
end for
$\widehat{\lambda}_1 \leftarrow \inf\{\lambda_1 \in \Lambda_1 : \bar{D}(\lambda_1) \leq \beta_1\}$
for λ_2 in Λ_2 **do**
    $\{\widehat{\boldsymbol{\Theta}}^{(k)}_{\widehat{\lambda}_1\lambda_2}, E_k\}_{k=1}^K \leftarrow \text{JGL}\left(\{\boldsymbol{X}^{(k)}\}_{k=1}^K \mid \widehat{\lambda}_1, \lambda_2\right)$
    $\text{BIC}_\gamma(\widehat{\lambda}_1,\lambda_2) \leftarrow \sum_{k=1}^K \left[n_k \text{tr}(\boldsymbol{S}^{(k)} \widehat{\boldsymbol{\Theta}}^{(k)}_{\widehat{\lambda}_1,\lambda_2}) - n_k \log(\det(\widehat{\boldsymbol{\Theta}}^{(k)}_{\widehat{\lambda}_1,\lambda_2})) + |E_k| \log n_k + 4|E_k|\gamma \log p\right]$
end for
$\widehat{\lambda}_2 \leftarrow \arg\min_{\lambda_2 \in \Lambda_2} \text{BIC}_\gamma(\widehat{\lambda}_1,\lambda_2)$
$\{\widehat{\boldsymbol{\Theta}}^{(k)}_{\text{stabJGL}}\}_{k=1}^K \leftarrow \text{JGL}\left(\{\boldsymbol{X}^{(k)}\}_{k=1}^K \mid \widehat{\lambda}_1, \widehat{\lambda}_2\right)$

50% of their edges in common, generated with the same approach as in the main simulation study. As discussed by Danaher et al., 2014, the group joint graphical lasso is faster that its fused counterpart. The Bayesian spike-and-slab joint graphical lasso is substantially slower than the other methods, taking around ten times longer than the fused joint graphical lasso and stabJGL. Figure S2 shows the time used by stabJGL to infer $K \in \{2, 3\}$ networks with various numbers of nodes p and 50% of their edges in common. We see that for $K = 2$ networks, inference for $p = 1{,}400$ nodes is feasible within half an hour, while for $K = 3$ inference for $p = 1{,}000$ nodes is feasible within about eight hours. As discussed by Danaher et al., 2014, there is an explicit solution to the fused joint graphical lasso problem for $K = 2$ and hence inference is much faster for stabJGL as well in that case. Additional results for up to $p = 2500$ nodes or up to $K = 10$ networks are shown in Figure S3.

S4 Choice of variability threshold

Figure S4 compares the performance of stabJGL for different values of the variability threshold β_1 to the the graphical lasso (Glasso), the fused joint graphical lasso (FGL), the group joint graphical lasso

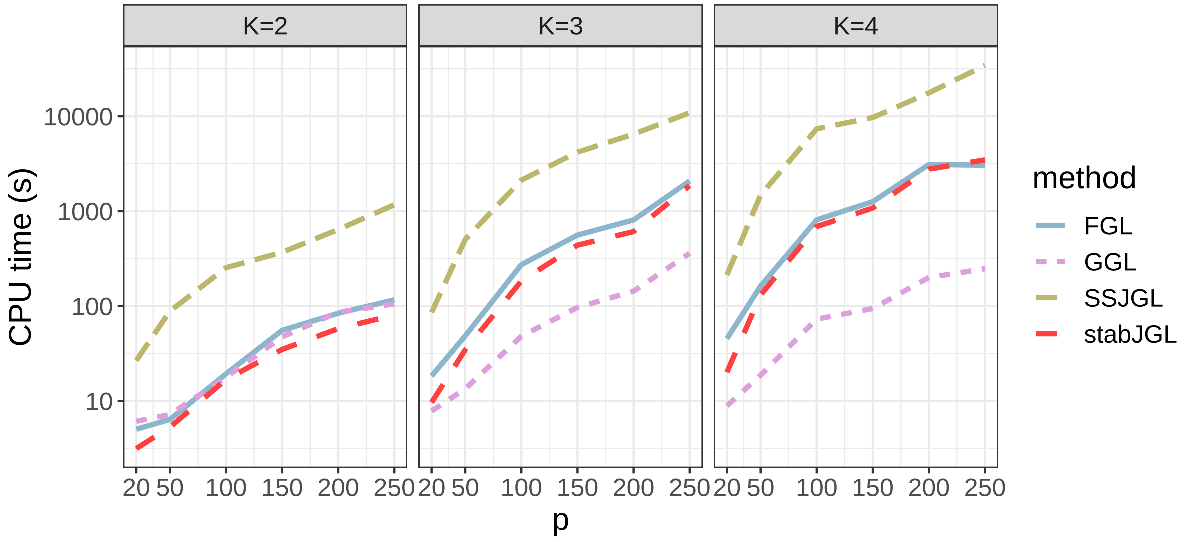

Figure S1: CPU time in seconds on a logarithmic scale used to jointly infer networks for $K \in \{2, 4\}$ networks and various numbers of nodes p, with $n \in \{100,150\}$ observations, for the fused and group joint graphical lasso with AIC penalty parameter selection (FGL and GGL), the Bayesian spike-and-slab joint graphical lasso (SSJGL) and stabJGL. The computations were performed on a 16-core Intel Xeon CPU, 2.60 GHz.

(GGL) and the Bayesian spike-and-slab joint graphical lasso (SSJGL). The results for FGL tuned with eBIC are not shown as it selected an empty graph in all settings. The settings considered have $K = 2$ networks with $p = 100$ nodes of various similarity. As in the setting considered in the main manuscript, we find that by varying the variability threshold β_1 we can obtain at least as high precision and/or recall as the other methods at any level of similarity.

S5 Order of penalty parameter selection

A relevant question is how the performance of the algorithm is affected by the order of which the penalty parameters λ_1 and λ_2 are selected. Table S7 compares the network reconstruction performance of the standard stabJGL algorithm with λ_1 selected before λ_2 to the stabJGL algorithm with λ_1 selected after λ_2. The settings considered are the same as in Table S1, with $K = 3$ networks and $p = 100$ nodes, and various similarity of the true graph structures, averaged over $N = 100$ simulations. It is clear that while the two approaches give similar results for networks with less similarity, the standard procedure of selecting λ_1 before λ_2 leads to a larger selected value of λ_1 and thus sparser network estimates for networks with higher similarity. This can be explained by the fact that for more similar networks, a larger λ_2 value is selected, thus leading to more edge selection stability because the networks are encouraged to be similar. Hence a smaller λ_1 is required to achieve edge selection stability. As a result, the standard approach leads to higher precision but lower recall than the alternative one. While achieving high precision and hence low discovery rates often is a desired feature in graph selection, one can choose to select λ_1 after λ_2 is high recall is favoured over high precision. This option is provided in the `stabJGL` R package.

S6 Effect of random noise on performance

It is also relevant to investigate the effect of random noise on the performance of stabJGL. Table S8 compares the network reconstruction performance of the different methods, considering the same settings as in Table S1, with $K = 3$ networks and $p = 100$ nodes, and various similarity of the true graph structures, averaged over $N = 100$ simulations. Random noise with mean 0 and a large standard deviation of 1 is added independently to each entry of the observation matrices $\boldsymbol{X}_k$, $k = 1, \ldots, K$. Not unexpectedly, comparing the results to those in Table S1 we see that all methods suffer from decreased

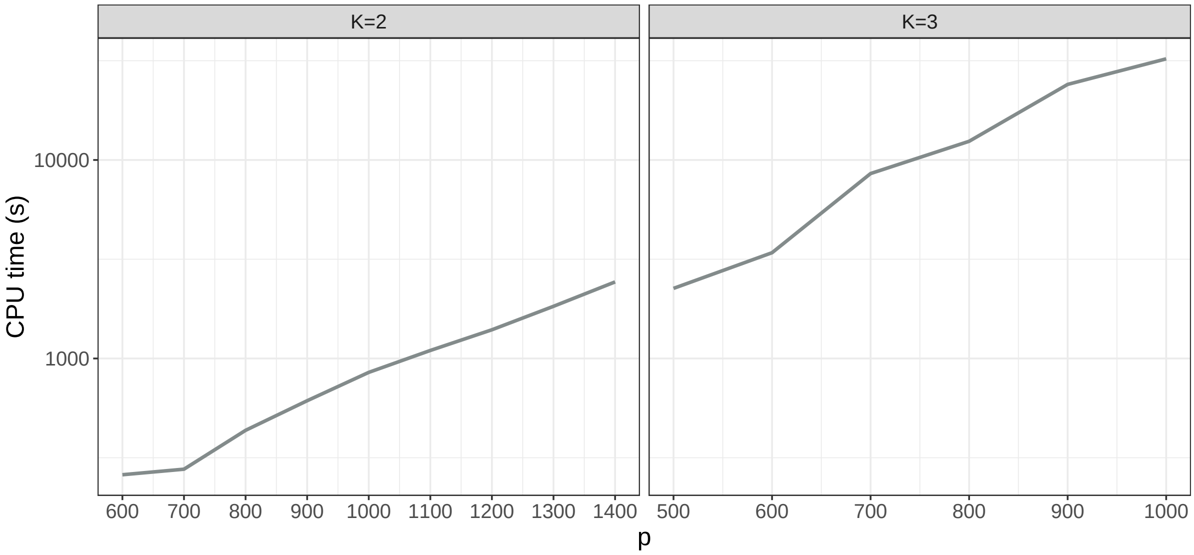

Figure S2: CPU time in seconds on a logarithmic scale used by stabJGL to jointly infer $K = 2$ networks with various numbers of nodes p from $n_1 = n_2 = 500$ observations and $K = 3$ networks with various numbers of nodes p from $n_1 = n_2 = n_3 = 500$ observations. The computations were performed on a 16-core Intel Xeon CPU, 2.60 GHz.

performance when large random noise is added. While the graphical lasso as well as FGL and GGL obtain very low precision in all settings, SSJGL and stabJGL obtain much higher precision though with lower recall. In all settings, however, stabJGL obtains comparable precision to SSJGL but with much higher recall. In the case of completely unrelated networks, only stabJGL achieves high precision. Overall, it appears that stabJGL adapts to increased noise in data by selecting fewer edges, hence maintaining high confidence.

S7 Additional Pan-Cancer analysis results

S7.1

A comparison of the pairwise network similarities is given in Figure S5, where similarity is measured by Matthew's Correlation Coefficient (MCC), a discretized Pearson correlation coefficient that can be used to quantify pairwise network similarities (Matthews, 1975). StabJGL finds the networks of the three tumor types to be more similar than FGL, in accordance with the findings of Akbani et al., 2014.

S7.2 Degree distributions

Table S6 shows the degree distribution of the proteomic networks identified by stabJGL and FGL. While the stabJGL networks all have degree distributions that follow clear power-law distributions, in line with biological expectations, the FGL networks have degree distributions that strongly contradict a power law with most nodes having node degree > 60.

S7.3 Top hubs

Table S9 shows the node degree of the proteins with degree larger than the 90th percentile in the respective stabJGL networks of the different tumor types. The same table for the FGL networks is shown in Table S9.

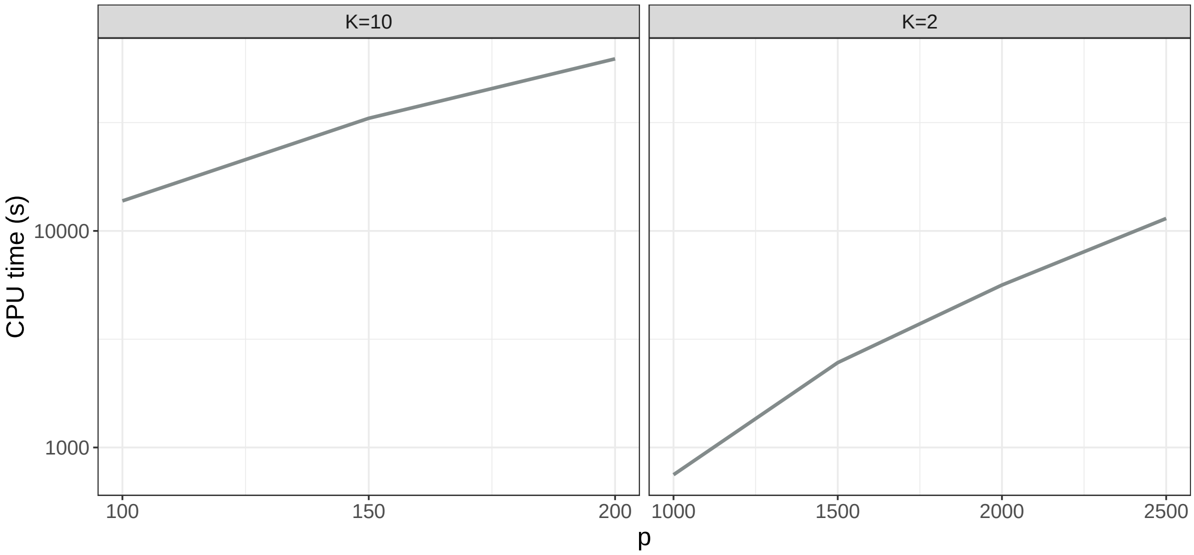

Figure S3: CPU time in seconds on a logarithmic scale used by stabJGL to jointly infer $K = 2$ networks with various numbers of nodes p from $n_1 = n_2 = 500$ observations and $K = 10$ networks with various numbers of nodes p from $n_k = 500$ observations, $k = 1, \ldots, 10$. The computations were performed on a 16-core Intel Xeon CPU, 2.60 GHz.

References

Akbani, Rehan et al. (2014). "A pan-cancer proteomic perspective on The Cancer Genome Atlas". In: *Nature communications* 5.1, p. 3887.

Danaher, Patrick, Wang, Pei, and Witten, Daniela M (2014). "The joint graphical lasso for inverse covariance estimation across multiple classes". In: *Journal of the Royal Statistical Society: Series B (Statistical Methodology)* 76.2, pp. 373–397.

Li, Zehang, Mccormick, Tyler, and Clark, Samuel (2019). "Bayesian joint spike-and-slab graphical lasso". *International Conference on Machine Learning*. PMLR, pp. 3877–3885.

Matthews, Brian W (1975). "Comparison of the predicted and observed secondary structure of T4 phage lysozyme". In: *Biochimica et Biophysica Acta (BBA)-Protein Structure* 405.2, pp. 442–451.

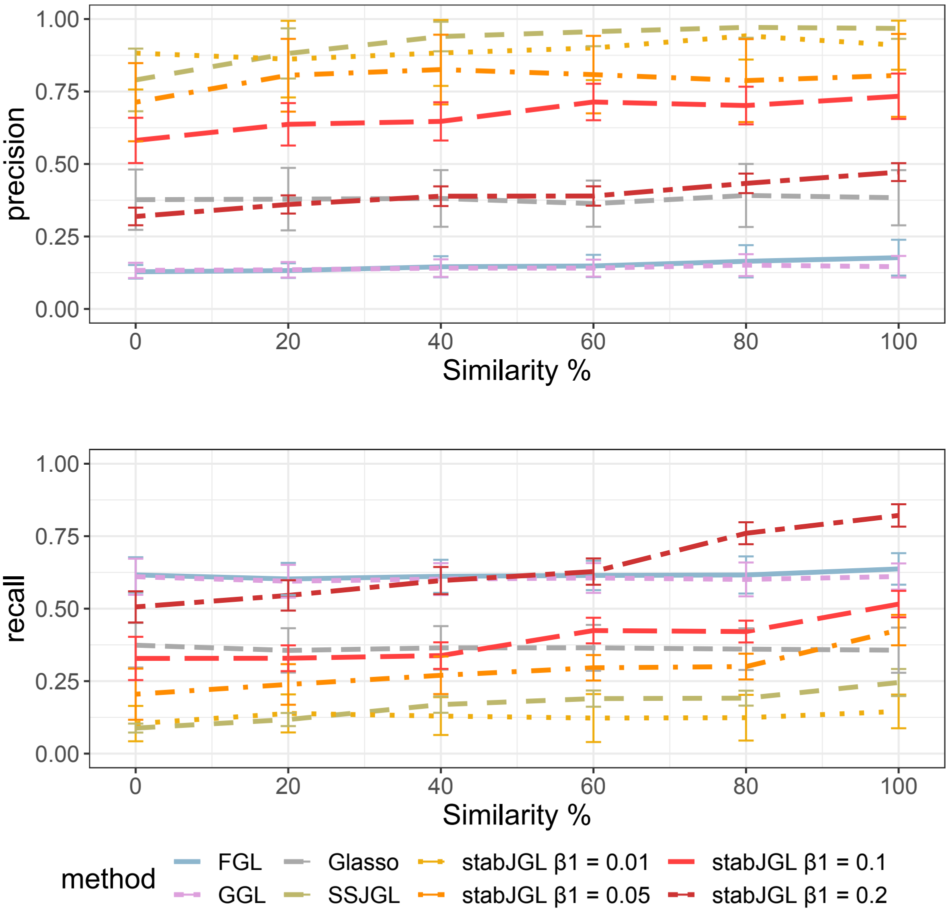

Figure S4: Performance of stabJGL for different values of the variability threshold β_1 on simulated data, compared to other graph reconstruction methods. The methods are used to estimate graphs with $p = 100$ nodes from $K = 2$ networks, both of sparsity 0.02, with various similarity of the true graph structures. The performance of stabJGL is compared to that of the graphical lasso (Glasso), the fused joint graphical lasso tuned by the AIC (FGL), the group joint graphical lasso (GGL) and the Bayesian spike-and-slab joint graphical lasso (SSJGL). The similarity (percentage of edges that are in common) of the graphs is shown. The results are averaged over $N = 100$ simulations and shows the precision and recall of each of the $K = 2$ estimated graphs. Standard deviation bars are shown for all methods. The graphs are reconstructed from $n_1 = 100$ and $n_2 = 150$ observations.

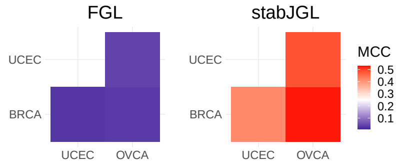

Figure S5: Pairwise Matthew's Correlation Coefficient between the proteomic network structures of the breast cancer (BRCA), ovarian cystadenocarcinoma (OVCA) and uterine corpus endometrial carcinoma (UCEC) tumors, identified by FGL tuned by the AIC and stabJGL respectively.

Table S1: Performance of the different graph reconstruction methods in simulations, reconstructing graphs with $p = 100$ nodes from $K = 3$ networks with various similarity of the true graph structures. The methods included are Glasso, FGL and GGL tuned by AIC, FGL tuned by eBIC, SSJGL and stabJGL. The similarity (percentage of edges that are in common) of the graphs is shown. The results are averaged over $N = 100$ simulations and shows the sparsity, precision, and recall of each of the $K = 3$ estimated graphs. The corresponding standard deviations are shown in parentheses. The graphs are reconstructed from $n_1 = 150$, $n_2 = 200$ and $n_3 = 300$ observations. All graphs have sparsity 0.02. The average selected values of the penalty parameters λ_1 and λ_2 for the relevant methods is shown as well.

				$n_1 = 150$			$n_2 = 200$			$n_3 = 300$		
Similarity	Method	λ_1	λ_2	Sparsity	Precision	Recall	Sparsity	Precision	Recall	Sparsity	Precision	Recall
100 %	Glasso	0.208	-	0.026 (0.007)	0.41 (0.08)	0.51 (0.06)	0.018 (0.004)	0.57 (0.08)	0.51 (0.05)	0.014 (0.002)	0.76 (0.07)	0.54 (0.05)
	FGL	0.114	0.021	0.087 (0.028)	0.22 (0.08)	0.88 (0.04)	0.064 (0.020)	0.31 (0.09)	0.90 (0.03)	0.041 (0.010)	0.46 (0.09)	0.91 (0.03)
	FGL (eBIC)	0.365	0.021	0.004 (0.004)	0.97 (0.06)	0.20 (0.20)	0.004 (0.004)	0.99 (0.03)	0.20 (0.20)	0.004 (0.004)	0.99 (0.01)	0.20 (0.20)
	GGL	0.114	0.007	0.152 (0.024)	0.11 (0.03)	0.81 (0.04)	0.114 (0.019)	0.15 (0.04)	0.84 (0.04)	0.069 (0.013)	0.27 (0.06)	0.87 (0.03)
	SSJGL	-	-	0.011 (0.001)	1.00 (0.00)	0.54 (0.04)	0.011 (0.001)	1.00 (0.00)	0.54 (0.04)	0.011 (0.001)	1.00 (0.00)	0.54 (0.04)
	stabJGL	0.166	0.067	0.015 (0.001)	0.88 (0.05)	0.66 (0.04)	0.015 (0.001)	0.90 (0.04)	0.66 (0.04)	0.015 (0.001)	0.91 (0.03)	0.66 (0.04)
80 %	Glasso	0.202	-	0.026 (0.007)	0.40 (0.08)	0.50 (0.06)	0.018 (0.005)	0.57 (0.12)	0.48 (0.07)	0.015 (0.002)	0.75 (0.07)	0.55 (0.06)
	FGL	0.114	0.015	0.105 (0.030)	0.18 (0.06)	0.84 (0.04)	0.072 (0.023)	0.25 (0.08)	0.82 (0.04)	0.045 (0.012)	0.41 (0.10)	0.86 (0.03)
	FGL (eBIC)	0.480	0.001	0.000 (0.000)	-	-	0.000 (0.000)	-	-	0.000 (0.000)	-	-
	GGL	0.115	0.008	0.149 (0.028)	0.11 (0.03)	0.80 (0.05)	0.107 (0.023)	0.16 (0.05)	0.80 (0.05)	0.065 (0.015)	0.28 (0.10)	0.85 (0.05)
	SSJGL	-	-	0.008 (0.001)	1.00 (0.01)	0.40 (0.04)	0.008 (0.001)	0.97 (0.03)	0.39 (0.04)	0.008 (0.001)	0.99 (0.01)	0.40 (0.04)
	stabJGL	0.166	0.053	0.014 (0.002)	0.84 (0.08)	0.59 (0.04)	0.012 (0.001)	0.90 (0.04)	0.54 (0.04)	0.012 (0.001)	0.93 (0.03)	0.56 (0.04)
60 %	Glasso	0.206	-	0.026 (0.007)	0.41 (0.08)	0.51 (0.06)	0.017 (0.005)	0.59 (0.11)	0.48 (0.08)	0.015 (0.002)	0.75 (0.06)	0.55 (0.05)
	FGL	0.114	0.010	0.124 (0.029)	0.14 (0.04)	0.81 (0.03)	0.086 (0.022)	0.20 (0.05)	0.81 (0.04)	0.054 (0.014)	0.33 (0.08)	0.84 (0.04)
	FGL (eBIC)	0.462	0.003	0.001 (0.003)	0.99 (0.05)	0.04 (0.12)	0.001 (0.002)	0.99 (0.02)	0.03 (0.10)	0.001 (0.002)	1.00 (0.01)	0.04 (0.11)
	GGL	0.114	0.006	0.156 (0.024)	0.11 (0.02)	0.80 (0.03)	0.112 (0.019)	0.15 (0.03)	0.81 (0.04)	0.070 (0.013)	0.25 (0.06)	0.85 (0.04)
	SSJGL	-	-	0.006 (0.001)	0.99 (0.02)	0.31 (0.03)	0.006 (0.001)	0.95 (0.03)	0.30 (0.03)	0.006 (0.001)	0.97 (0.03)	0.31 (0.03)
	stabJGL	0.166	0.044	0.015 (0.003)	0.75 (0.09)	0.55 (0.05)	0.012 (0.001)	0.87 (0.05)	0.50 (0.04)	0.011 (0.001)	0.92 (0.04)	0.52 (0.04)
40 %	Glasso	0.202	-	0.027 (0.008)	0.39 (0.07)	0.51 (0.06)	0.018 (0.005)	0.57 (0.10)	0.49 (0.07)	0.015 (0.003)	0.77 (0.09)	0.55 (0.07)
	FGL	0.114	0.007	0.137 (0.024)	0.12 (0.03)	0.80 (0.04)	0.097 (0.021)	0.17 (0.04)	0.80 (0.04)	0.055 (0.013)	0.32 (0.07)	0.84 (0.05)
	FGL (eBIC)	0.485	0.001	0.000 (0.000)	-	-	0.000 (0.000)	-	-	0.000 (0.000)	-	-
	GGL	0.114	0.004	0.158 (0.018)	0.10 (0.02)	0.81 (0.04)	0.115 (0.016)	0.14 (0.02)	0.81 (0.04)	0.067 (0.010)	0.26 (0.05)	0.85 (0.04)
	SSJGL	-	-	0.004 (0.001)	0.88 (0.06)	0.16 (0.03)	0.004 (0.001)	0.82 (0.07)	0.15 (0.03)	0.004 (0.001)	0.90 (0.06)	0.16 (0.03)
	stabJGL	0.166	0.038	0.016 (0.003)	0.64 (0.09)	0.51 (0.04)	0.011 (0.001)	0.83 (0.06)	0.46 (0.04)	0.009 (0.001)	0.93 (0.03)	0.44 (0.04)
20 %	Glasso	0.205	-	0.026 (0.007)	0.40 (0.08)	0.51 (0.06)	0.018 (0.004)	0.59 (0.10)	0.51 (0.07)	0.015 (0.002)	0.75 (0.06)	0.55 (0.05)
	FGL	0.114	0.003	0.154 (0.018)	0.10 (0.01)	0.79 (0.04)	0.112 (0.016)	0.15 (0.02)	0.82 (0.04)	0.068 (0.010)	0.26 (0.04)	0.86 (0.04)
	FGL (eBIC)	0.482	0.001	0.000 (0.000)	-	-	0.000 (0.000)	-	-	0.000 (0.00)	-	-
	GGL	0.114	0.002	0.164 (0.011)	0.10 (0.01)	0.80 (0.04)	0.121 (0.010)	0.14 (0.01)	0.83 (0.03)	0.073 (0.007)	0.24 (0.02)	0.87 (0.03)
	SSJGL	-	-	0.003 (0.001)	0.83 (0.07)	0.12 (0.03)	0.003 (0.001)	0.81 (0.08)	0.12 (0.02)	0.003 (0.001)	0.90 (0.07)	0.13 (0.03)
	stabJGL	0.166	0.036	0.016 (0.003)	0.61 (0.08)	0.48 (0.05)	0.012 (0.002)	0.79 (0.07)	0.46 (0.05)	0.010 (0.001)	0.92 (0.04)	0.45 (0.04)
0 %	Glasso	0.204	-	0.027 (0.008)	0.40 (0.08)	0.51 (0.06)	0.020 (0.005)	0.69 (0.10)	0.66 (0.08)	0.019 (0.004)	0.83 (0.08)	0.77 (0.08)
	FGL	0.114	0.001	0.165 (0.011)	0.10 (0.01)	0.81 (0.04)	0.118 (0.010)	0.16 (0.02)	0.94 (0.02)	0.069 (0.006)	0.28 (0.03)	0.97 (0.02)
	FGL (eBIC)	0.446	0.002	0.002 (0.003)	0.96 (0.09)	0.06 (0.12)	0.001 (0.003)	0.99 (0.03)	0.07 (0.13)	0.001 (0.003)	1.00 (0.01)	0.07 (0.14)
	GGL	0.114	0.000	0.168 (0.007)	0.10 (0.00)	0.81 (0.04)	0.121 (0.006)	0.16 (0.01)	0.94 (0.02)	0.071 (0.004)	0.28 (0.02)	0.97 (0.02)
	SSJGL	-	-	0.002 (0.001)	0.44 (0.11)	0.05 (0.02)	0.003 (0.001)	0.70 (0.12)	0.09 (0.02)	0.003 (0.001)	0.73 (0.10)	0.09 (0.02)
	stabJGL	0.166	0.024	0.025 (0.006)	0.43 (0.07)	0.51 (0.05)	0.018 (0.003)	0.73 (0.07)	0.64 (0.07)	0.015 (0.002)	0.90 (0.04)	0.66 (0.08)

Table S2: Performance of the different graph reconstruction methods in simulations, reconstructing graphs with $p = 200$ nodes from $K = 3$ classes with various similarity of the true graph structures. The methods included are the graphical lasso (Glasso), the fused joint graphical lasso tuned by the AIC (FGL) and by the extended BIC (eBIC), the group joint graphical lasso (GGL), the Bayesian spike-and-slab joint graphical lasso (SSJGL) and stabJGL. The similarity (percentage of edges that are in common) of the graphs is shown. The results are averaged over $N = 100$ simulations and shows the sparsity, precision, and recall of each of the $K = 3$ estimated graphs. The corresponding standard deviations are shown as well. The graphs are reconstructed from $n_1 = 150$, $n_2 = 200$ and $n_3 = 300$ observations. All graphs have sparsity 0.01. The average selected values of the penalty parameters λ_1 and λ_2 for the relevant methods is shown as well.

				$n_1 = 150$			$n_2 = 200$			$n_3 = 300$		
Similarity	Method	λ_1	λ_2	Sparsity	Precision	Recall	Sparsity	Precision	Recall	Sparsity	Precision	Recall
100 %	Glasso	0.201	-	0.018 (0.007)	0.22 (0.05)	0.37 (0.05)	0.010 (0.004)	0.40 (0.09)	0.37 (0.05)	0.006 (0.002)	0.64 (0.09)	0.39 (0.05)
	FGL	0.166	0.013	0.027 (0.012)	0.21 (0.11)	0.49 (0.04)	0.015 (0.006)	0.38 (0.15)	0.48 (0.03)	0.007 (0.002)	0.68 (0.14)	0.46 (0.04)
	FGL (eBIC)	0.488	0.000	0.000 (0.000)	-	-	0.000 (0.000)	-	-	0.000 (0.000)	-	-
	GGL	0.166	0.004	0.044 (0.006)	0.12 (0.02)	0.50 (0.04)	0.024 (0.004)	0.21 (0.04)	0.49 (0.03)	0.010 (0.002)	0.48 (0.06)	0.48 (0.04)
	SSJGL	-	-	0.002 (0.000)	1.00 (0.00)	0.25 (0.03)	0.002 (0.000)	1.00 (0.00)	0.25 (0.03)	0.002 (0.000)	1.00 (0.00)	0.25 (0.03)
	stabJGL	0.166	0.078	0.005 (0.000)	0.89 (0.05)	0.44 (0.02)	0.005 (0.000)	0.90 (0.03)	0.44 (0.02)	0.005 (0.000)	0.91 (0.03)	0.44 (0.02)
80 %	Glasso	0.200	-	0.017 (0.006)	0.23 (0.06)	0.36 (0.05)	0.010 (0.003)	0.40 (0.09)	0.38 (0.05)	0.006 (0.002)	0.63 (0.10)	0.38 (0.05)
	FGL	0.166	0.005	0.039 (0.011)	0.14 (0.05)	0.48 (0.04)	0.021 (0.006)	0.25 (0.08)	0.49 (0.04)	0.008 (0.002)	0.56 (0.13)	0.45 (0.04)
	FGL (eBIC)	0.489	0.000	0.000 (0.000)	-	-	0.000 (0.000)	-	-	0.000 (0.000)	-	-
	GGL	0.166	0.003	0.045 (0.005)	0.11 (0.01)	0.49 (0.03)	0.025 (0.003)	0.21 (0.03)	0.50 (0.04)	0.010 (0.001)	0.49 (0.06)	0.46 (0.03)
	SSJGL	-	-	0.002 (0.000)	1.00 (0.00)	0.17 (0.02)	0.002 (0.000)	0.98 (0.02)	0.17 (0.02)	0.002 (0.000)	0.99 (0.01)	0.17 (0.02)
	stabJGL	0.166	0.064	0.005 (0.001)	0.84 (0.07)	0.37 (0.03)	0.004 (0.000)	0.88 (0.04)	0.35 (0.03)	0.004 (0.000)	0.91 (0.04)	0.34 (0.03)
60 %	Glasso	0.196	-	0.018 (0.006)	0.22 (0.06)	0.37 (0.05)	0.011 (0.004)	0.37 (0.10)	0.36 (0.05)	0.007 (0.002)	0.60 (0.10)	0.41 (0.06)
	FGL	0.166	0.003	0.043 (0.008)	0.12 (0.03)	0.50 (0.04)	0.022 (0.005)	0.22 (0.05)	0.47 (0.04)	0.009 (0.002)	0.52 (0.08)	0.46 (0.04)
	FGL (eBIC)	0.484	0.000	0.000 (0.000)	-	-	0.000 (0.000)	-	-	0.000 (0.000)	-	-
	GGL	0.166	0.003	0.045 (0.005)	0.11 (0.01)	0.50 (0.04)	0.024 (0.003)	0.20 (0.02)	0.47 (0.03)	0.009 (0.001)	0.50 (0.05)	0.46 (0.04)
	SSJGL	-	-	0.001 (0.000)	1.00 (0.01)	0.10 (0.02)	0.001 (0.000)	0.95 (0.05)	0.10 (0.02)	0.001 (0.000)	0.99 (0.02)	0.10 (0.02)
	stabJGL	0.166	0.056	0.004 (0.001)	0.77 (0.07)	0.34 (0.03)	0.003 (0.000)	0.85 (0.04)	0.29 (0.03)	0.003 (0.000)	0.92 (0.03)	0.29 (0.03)
40 %	Glasso	0.199	-	0.017 (0.006)	0.23 (0.05)	0.36 (0.05)	0.011 (0.004)	0.38 (0.11)	0.36 (0.06)	0.006 (0.002)	0.63 (0.13)	0.37 (0.07)
	FGL	0.166	0.002	0.046 (0.008)	0.11 (0.02)	0.50 (0.04)	0.024 (0.005)	0.21 (0.04)	0.48 (0.04)	0.009 (0.002)	0.50 (0.07)	0.44 (0.04)
	FGL (eBIC)	0.482	0.000	0.000 (0.000)	-	-	0.000 (0.000)	-	-	0.000 (0.000)	-	-
	GGL	0.166	0.002	0.046 (0.005)	0.11 (0.01)	0.50 (0.03)	0.024 (0.003)	0.20 (0.02)	0.48 (0.04)	0.009 (0.001)	0.49 (0.05)	0.44 (0.03)
	SSJGL	-	-	0.001 (0.000)	0.98 (0.04)	0.06 (0.01)	0.001 (0.000)	0.92 (0.07)	0.05 (0.01)	0.001 (0.000)	0.98 (0.04)	0.06 (0.01)
	stabJGL	0.166	0.057	0.004 (0.001)	0.75 (0.09)	0.28 (0.03)	0.003 (0.000)	0.83 (0.05)	0.23 (0.03)	0.002 (0.000)	0.91 (0.04)	0.23 (0.03)
20 %	Glasso	0.198	-	0.019 (0.007)	0.22 (0.06)	0.38 (0.05)	0.010 (0.004)	0.38 (0.11)	0.34 (0.07)	0.006 (0.002)	0.63 (0.11)	0.39 (0.06)
	FGL	0.166	0.001	0.047 (0.005)	0.11 (0.01)	0.50 (0.03)	0.024 (0.003)	0.20 (0.03)	0.47 (0.04)	0.010 (0.001)	0.48 (0.06)	0.47 (0.03)
	FGL (eBIC)	0.486	0.000	0.000 (0.000)	-	-	0.000 (0.000)	-	-	0.000 (0.000)	-	-
	GGL	0.166	0.002	0.046 (0.005)	0.11 (0.01)	0.50 (0.03)	0.024 (0.003)	0.20 (0.03)	0.47 (0.04)	0.010 (0.001)	0.48 (0.05)	0.47 (0.03)
	SSJGL	-	-	0.000 (0.000)	0.90 (0.12)	0.03 (0.01)	0.000 (0.000)	0.87 (0.12)	0.03 (0.01)	0.000 (0.000)	0.96 (0.07)	0.03 (0.01)
	stabJGL	0.166	0.052	0.004 (0.001)	0.65 (0.09)	0.27 (0.03)	0.003 (0.000)	0.82 (0.05)	0.22 (0.03)	0.002 (0.000)	0.91 (0.04)	0.22 (0.03)
0 %	Glasso	0.201	-	0.017 (0.005)	0.23 (0.05)	0.36 (0.04)	0.011 (0.004)	0.54 (0.12)	0.57 (0.08)	0.008 (0.003)	0.73 (0.12)	0.52 (0.09)
	FGL	0.140	0.005	0.077 (0.031)	0.08 (0.02)	0.56 (0.07)	0.048 (0.023)	0.20 (0.08)	0.79 (0.06)	0.023 (0.013)	0.40 (0.16)	0.73 (0.09)
	FGL (eBIC)	0.497	0.001	0.000 (0.000)	-	-	0.000 (0.000)	-	-	0.000 (0.000)	-	-
	GGL	0.166	0.000	0.048 (0.002)	0.10 (0.01)	0.50 (0.03)	0.027 (0.001)	0.28 (0.01)	0.74 (0.03)	0.012 (0.001)	0.56 (0.03)	0.65 (0.03)
	SSJGL	-	-	0.000 (0.000)	0.47 (0.21)	0.01 (0.01)	0.000 (0.000)	0.79 (0.18)	0.02 (0.01)	0.000 (0.000)	0.84 (0.17)	0.02 (0.01)
	stabJGL	0.166	0.047	0.005 (0.001)	0.53 (0.09)	0.23 (0.04)	0.004 (0.001)	0.84 (0.05)	0.31 (0.06)	0.003 (0.000)	0.92 (0.04)	0.24 (0.04)

Table S3: Performance of the different graph reconstruction methods in simulations, reconstructing graphs with $p = 100$ nodes from $K = 4$ classes with various similarity of the true graph structures. The methods included are the graphical lasso (Glasso), the fused joint graphical lasso tuned by the AIC (FGL) and by the extended BIC (eBIC), the group joint graphical lasso (GGL), the Bayesian spike-and-slab joint graphical lasso (SSJGL) and stabJGL. The similarity (percentage of edges that are in common) of the graphs is shown. The results are averaged over $N = 100$ simulations and shows the sparsity, precision, and recall of each of the $K = 4$ estimated graphs. The corresponding standard deviations are shown as well. The graphs are reconstructed from $n_1 = 150$, $n_2 = 200$, $n_3 = 250$ and $n_4 = 300$ observations. All graphs have sparsity 0.02. The average selected values of the penalty parameters λ_1 and λ_2 for the relevant methods is shown as well.

				$n_1 = 150$			$n_2 = 200$			$n_3 = 250$			$n_4 = 300$		
Similarity	Method	λ_1	λ_2	Sparsity	Precision	Recall	Sparsity	Precision	Recall	Sparsity	Precision	Recall	Sparsity	Precision	Recall
100 %	Glasso	0.206	-	0.026 (0.007)	0.40 (0.08)	0.51 (0.06)	0.018 (0.005)	0.57 (0.09)	0.51 (0.06)	0.016 (0.003)	0.69 (0.08)	0.53 (0.06)	0.015 (0.003)	0.75 (0.08)	0.55 (0.06)
	FGL	0.114	0.02	0.065 (0.028)	0.33 (0.15)	0.90 (0.04)	0.047 (0.020)	0.44 (0.15)	0.92 (0.03)	0.038 (0.014)	0.53 (0.14)	0.92 (0.04)	0.033 (0.010)	0.61 (0.13)	0.93 (0.03)
	FGL (eBIC)	0.232	0.036	0.008 (0.002)	0.98 (0.04)	0.38 (0.09)	0.008 (0.002)	0.99 (0.02)	0.38 (0.09)	0.008 (0.002)	1.00 (0.01)	0.38 (0.09)	0.007 (0.002)	1.00 (0.00)	0.37 (0.09)
	GGL	0.114	0.002	0.165 (0.014)	0.10 (0.02)	0.80 (0.04)	0.123 (0.011)	0.14 (0.02)	0.84 (0.04)	0.095 (0.010)	0.18 (0.04)	0.86 (0.04)	0.075 (0.007)	0.24 (0.03)	0.88 (0.04)
	SSJGL	-	-	0.012 (0.001)	1.00 (0.00)	0.61 (0.04)	0.012 (0.001)	1.00 (0.00)	0.61 (0.04)	0.012 (0.001)	1.00 (0.00)	0.61 (0.04)	0.012 (0.001)	1.00 (0.00)	0.61 (0.04)
	stabJGL	0.166	0.042	0.014 (0.002)	0.92 (0.07)	0.66 (0.04)	0.014 (0.001)	0.95 (0.04)	0.66 (0.04)	0.014 (0.001)	0.97 (0.02)	0.66 (0.04)	0.014 (0.001)	0.97 (0.02)	0.66 (0.04)
80 %	Glasso	0.201	-	0.026 (0.007)	0.41 (0.09)	0.50 (0.05)	0.018 (0.005)	0.57 (0.12)	0.49 (0.07)	0.016 (0.003)	0.70 (0.09)	0.54 (0.07)	0.015 (0.003)	0.75 (0.07)	0.55 (0.06)
	FGL	0.114	0.012	0.091 (0.025)	0.20 (0.06)	0.85 (0.04)	0.061 (0.019)	0.30 (0.09)	0.83 (0.04)	0.047 (0.015)	0.40 (0.11)	0.86 (0.03)	0.038 (0.010)	0.48 (0.11)	0.87 (0.03)
	FGL (eBIC)	0.407	0.008	0.003 (0.004)	0.97 (0.06)	0.13 (0.18)	0.002 (0.003)	0.99 (0.02)	0.11 (0.16)	0.002 (0.003)	1.00 (0.01)	0.12 (0.17)	0.002 (0.003)	1.00 (0.00)	0.12 (0.17)
	GGL	0.114	0.003	0.161 (0.019)	0.10 (0.02)	0.80 (0.04)	0.117 (0.016)	0.14 (0.04)	0.81 (0.04)	0.090 (0.012)	0.19 (0.04)	0.84 (0.03)	0.072 (0.010)	0.24 (0.05)	0.86 (0.04)
	SSJGL	-	-	0.010 (0.001)	1.00 (0.00)	0.49 (0.04)	0.010 (0.001)	0.94 (0.02)	0.45 (0.03)	0.010 (0.001)	0.96 (0.02)	0.46 (0.04)	0.010 (0.001)	0.96 (0.02)	0.47 (0.04)
	stabJGL	0.166	0.031	0.015 (0.002)	0.81 (0.11)	0.58 (0.04)	0.012 (0.001)	0.92 (0.05)	0.54 (0.04)	0.012 (0.001)	0.96 (0.03)	0.56 (0.04)	0.011 (0.001)	0.97 (0.02)	0.55 (0.04)
60 %	Glasso	0.203	-	0.026 (0.007)	0.40 (0.07)	0.51 (0.06)	0.018 (0.005)	0.57 (0.10)	0.49 (0.07)	0.015 (0.003)	0.70 (0.08)	0.53 (0.05)	0.014 (0.003)	0.75 (0.08)	0.53 (0.06)
	FGL	0.114	0.006	0.127 (0.030)	0.14 (0.04)	0.82 (0.04)	0.087 (0.024)	0.20 (0.06)	0.80 (0.04)	0.068 (0.019)	0.27 (0.08)	0.83 (0.04)	0.052 (0.015)	0.35 (0.10)	0.83 (0.04)
	FGL (eBIC)	0.435	0.005	0.002 (0.004)	0.97 (0.07)	0.08 (0.16)	0.002 (0.003)	0.99 (0.04)	0.07 (0.14)	0.002 (0.003)	0.99 (0.02)	0.08 (0.15)	0.001 (0.003)	1.00 (0.01)	0.07 (0.14)
	GGL	0.114	0.000	0.169 (0.006)	0.10 (0.01)	0.81 (0.04)	0.122 (0.006)	0.13 (0.01)	0.81 (0.04)	0.095 (0.005)	0.18 (0.01)	0.85 (0.03)	0.074 (0.004)	0.23 (0.02)	0.85 (0.04)
	SSJGL	-	-	0.007 (0.001)	0.98 (0.03)	0.32 (0.03)	0.007 (0.001)	0.90 (0.04)	0.30 (0.03)	0.007 (0.001)	0.89 (0.04)	0.29 (0.03)	0.007 (0.001)	0.86 (0.04)	0.28 (0.03)
	stabJGL	0.166	0.025	0.017 (0.003)	0.68 (0.09)	0.55 (0.04)	0.012 (0.001)	0.87 (0.06)	0.50 (0.04)	0.011 (0.001)	0.92 (0.04)	0.52 (0.05)	0.010 (0.001)	0.95 (0.03)	0.49 (0.05)
40 %	Glasso	0.202	-	0.026 (0.007)	0.41 (0.08)	0.50 (0.06)	0.017 (0.005)	0.59 (0.11)	0.47 (0.08)	0.015 (0.004)	0.71 (0.11)	0.52 (0.07)	0.014 (0.003)	0.78 (0.09)	0.52 (0.08)
	FGL	0.114	0.003	0.146 (0.025)	0.11 (0.02)	0.80 (0.04)	0.104 (0.021)	0.16 (0.03)	0.79 (0.04)	0.078 (0.018)	0.23 (0.06)	0.83 (0.04)	0.059 (0.014)	0.30 (0.07)	0.83 (0.04)
	FGL (eBIC)	0.489	0.000	0.000 (0.000)	1.00 (0.00)	0.00 (0.01)	0.000 (0.000)	1.00 (0.00)	0.00 (0.00)	0.000 (0.000)	1.00 (0.00)	0.00 (0.00)	0.000 (0.000)	1.00 (0.00)	0.00 (0.00)
	GGL	0.114	0.000	0.169 (0.005)	0.10 (0.00)	0.81 (0.04)	0.123 (0.005)	0.13 (0.01)	0.81 (0.04)	0.093 (0.005)	0.18 (0.01)	0.84 (0.03)	0.071 (0.004)	0.24 (0.01)	0.85 (0.03)
	SSJGL	-	-	0.004 (0.001)	0.93 (0.05)	0.19 (0.03)	0.004 (0.001)	0.69 (0.07)	0.14 (0.03)	0.004 (0.001)	0.77 (0.08)	0.15 (0.03)	0.004 (0.001)	0.81 (0.07)	0.16 (0.03)
	stabJGL	0.166	0.025	0.017 (0.004)	0.63 (0.10)	0.50 (0.05)	0.011 (0.002)	0.83 (0.07)	0.43 (0.05)	0.010 (0.001)	0.91 (0.04)	0.44 (0.05)	0.009 (0.001)	0.96 (0.03)	0.41 (0.05)
20 %	Glasso	0.203	-	0.026 (0.007)	0.41 (0.08)	0.50 (0.06)	0.018 (0.004)	0.58 (0.09)	0.51 (0.06)	0.016 (0.003)	0.67 (0.07)	0.54 (0.06)	0.015 (0.003)	0.76 (0.09)	0.54 (0.07)
	FGL	0.114	0.002	0.154 (0.022)	0.11 (0.02)	0.80 (0.04)	0.113 (0.019)	0.15 (0.03)	0.82 (0.04)	0.085 (0.015)	0.21 (0.04)	0.84 (0.03)	0.065 (0.013)	0.27 (0.05)	0.85 (0.05)
	FGL (eBIC)	0.450	0.003	0.002 (0.004)	0.95 (0.11)	0.06 (0.14)	0.001 (0.003)	0.98 (0.04)	0.06 (0.14)	0.001 (0.003)	0.99 (0.02)	0.06 (0.14)	0.001 (0.003)	1.00 (0.01)	0.06 (0.13)
	GGL	0.114	0.000	0.169 (0.005)	0.10 (0.00)	0.81 (0.04)	0.125 (0.004)	0.13 (0.01)	0.84 (0.04)	0.095 (0.004)	0.18 (0.01)	0.86 (0.03)	0.074 (0.004)	0.23 (0.02)	0.86 (0.04)
	SSJGL	-	-	0.004 (0.000)	0.79 (0.07)	0.15 (0.02)	0.004 (0.000)	0.68 (0.07)	0.13 (0.02)	0.004 (0.001)	0.79 (0.06)	0.15 (0.02)	0.004 (0.001)	0.82 (0.06)	0.16 (0.02)
	stabJGL	0.166	0.023	0.017 (0.004)	0.59 (0.09)	0.48 (0.05)	0.012 (0.002)	0.78 (0.08)	0.46 (0.05)	0.010 (0.001)	0.89 (0.06)	0.46 (0.04)	0.009 (0.001)	0.96 (0.04)	0.42 (0.04)
0 %	Glasso	0.207	-	0.027 (0.008)	0.40 (0.08)	0.51 (0.07)	0.019 (0.005)	0.70 (0.10)	0.65 (0.08)	0.018 (0.004)	0.80 (0.09)	0.70 (0.08)	0.015 (0.003)	0.85 (0.07)	0.63 (0.07)
	FGL	0.114	0.000	0.170 (0.005)	0.10 (0.00)	0.81 (0.04)	0.122 (0.004)	0.16 (0.01)	0.94 (0.02)	0.092 (0.004)	0.21 (0.01)	0.96 (0.02)	0.072 (0.003)	0.26 (0.01)	0.95 (0.02)
	FGL (eBIC)	0.380	0.011	0.004 (0.005)	0.91 (0.13)	0.14 (0.16)	0.003 (0.004)	0.98 (0.04)	0.15 (0.18)	0.003 (0.004)	0.99 (0.04)	0.15 (0.19)	0.002 (0.003)	0.99 (0.02)	0.12 (0.15)
	GGL	0.114	0.000	0.170 (0.005)	0.10 (0.00)	0.81 (0.04)	0.122 (0.004)	0.16 (0.01)	0.94 (0.02)	0.092 (0.004)	0.21 (0.01)	0.96 (0.02)	0.072 (0.003)	0.26 (0.01)	0.95 (0.02)
	SSJGL	-	-	0.003 (0.000)	0.59 (0.10)	0.07 (0.02)	0.002 (0.000)	0.63 (0.10)	0.08 (0.02)	0.002 (0.000)	0.48 (0.09)	0.06 (0.01)	0.003 (0.000)	0.69 (0.10)	0.09 (0.02)
	stabJGL	0.166	0.015	0.026 (0.006)	0.42 (0.07)	0.52 (0.06)	0.018 (0.004)	0.73 (0.08)	0.65 (0.07)	0.016 (0.003)	0.85 (0.06)	0.66 (0.07)	0.013 (0.002)	0.89 (0.05)	0.57 (0.08)

Table S4: Performance of the different graph reconstruction methods in simulations, reconstructing graphs with $p = 100$ nodes from $K = 2$ classes with various similarity of the true graph structures. The methods included are the graphical lasso (Glasso), the fused joint graphical lasso tuned by the AIC (FGL) and by the extended BIC (eBIC), the group joint graphical lasso (GGL), the Bayesian spike-and-slab joint graphical lasso (SSJGL) and stabJGL. The similarity (percentage of edges that are in common) of the graphs is shown. The results are averaged over $N = 100$ simulations and shows the sparsity, precision, and recall of each of the $K = 2$ estimated graphs. The corresponding standard deviations are shown as well. The graphs are reconstructed from $n_1 = 100$ and $n_2 = 150$ observations. All graphs have sparsity 0.02. The average selected values of the penalty parameters λ_1 and λ_2 for the relevant methods is shown as well.

				$n_1 = 100$			$n_2 = 150$		
Similarity	Method	λ_1	λ_2	Sparsity	Precision	Recall	Sparsity	Precision	Recall
100 %	Glasso	0.241	-	0.021 (0.009)	0.38 (0.10)	0.36 (0.08)	0.024 (0.006)	0.43 (0.08)	0.50 (0.05)
	FGL	0.168	0.022	0.079 (0.023)	0.18 (0.06)	0.64 (0.05)	0.045 (0.013)	0.31 (0.09)	0.65 (0.05)
	FGL (eBIC)	0.512	0.001	0.000 (0.000)	-	-	0.000 (0.000)	-	-
	GGL	0.167	0.014	0.088 (0.019)	0.15 (0.04)	0.61 (0.05)	0.049 (0.013)	0.28 (0.08)	0.63 (0.05)
	SSJGL	-	-	0.005 (0.001)	0.97 (0.04)	0.25 (0.05)	0.005 (0.001)	0.97 (0.04)	0.25 (0.05)
	stabJGL	0.218	0.095	0.014 (0.002)	0.69 (0.06)	0.47 (0.04)	0.012 (0.001)	0.78 (0.06)	0.47 (0.03)
80 %	Glasso	0.238	-	0.021 (0.009)	0.39 (0.11)	0.36 (0.07)	0.024 (0.008)	0.40 (0.10)	0.45 (0.07)
	FGL	0.170	0.017	0.082 (0.023)	0.16 (0.06)	0.62 (0.06)	0.042 (0.012)	0.31 (0.11)	0.58 (0.07)
	FGL (eBIC)	0.504	0.002	0.000 (0.000)	-	-	0.000 (0.000)	-	-
	GGL	0.169	0.014	0.084 (0.020)	0.15 (0.04)	0.60 (0.06)	0.042 (0.012)	0.29 (0.08)	0.56 (0.07)
	SSJGL	-	-	0.004 (0.001)	0.97 (0.04)	0.19 (0.03)	0.004 (0.001)	0.97 (0.04)	0.19 (0.03)
	stabJGL	0.218	0.093	0.012 (0.002)	0.69 (0.07)	0.40 (0.04)	0.009 (0.001)	0.82 (0.05)	0.37 (0.04)
60 %	Glasso	0.236	-	0.022 (0.008)	0.36 (0.08)	0.36 (0.08)	0.026 (0.007)	0.39 (0.07)	0.48 (0.07)
	FGL	0.168	0.013	0.087 (0.019)	0.15 (0.04)	0.61 (0.05)	0.048 (0.011)	0.26 (0.06)	0.61 (0.06)
	FGL (eBIC)	0.510	0.001	0.000 (0.000)	-	-	0.000 (0.000)	-	-
	GGL	0.167	0.012	0.089 (0.017)	0.14 (0.03)	0.61 (0.05)	0.049 (0.010)	0.26 (0.05)	0.60 (0.06)
	SSJGL	-	-	0.004 (0.001)	0.96 (0.05)	0.19 (0.03)	0.004 (0.001)	0.96 (0.04)	0.19 (0.03)
	stabJGL	0.218	0.092	0.012 (0.002)	0.66 (0.07)	0.39 (0.04)	0.010 (0.001)	0.78 (0.07)	0.38 (0.03)
40 %	Glasso	0.239	-	0.021 (0.009)	0.38 (0.10)	0.36 (0.08)	0.025 (0.007)	0.41 (0.08)	0.50 (0.06)
	FGL	0.168	0.013	0.089 (0.020)	0.15 (0.04)	0.61 (0.06)	0.049 (0.012)	0.27 (0.07)	0.62 (0.06)
	FGL (eBIC)	0.519	0.001	0.000 (0.000)	-	-	0.000 (0.000)	-	-
	GGL	0.167	0.012	0.089 (0.019)	0.14 (0.03)	0.60 (0.05)	0.049 (0.012)	0.26 (0.06)	0.61 (0.07)
	SSJGL	-	-	0.004 (0.001)	0.94 (0.05)	0.17 (0.03)	0.004 (0.001)	0.96 (0.05)	0.17 (0.03)
	stabJGL	0.218	0.092	0.011 (0.002)	0.66 (0.07)	0.36 (0.04)	0.009 (0.001)	0.79 (0.05)	0.35 (0.04)
20 %	Glasso	0.239	-	0.021 (0.009)	0.38 (0.11)	0.36 (0.08)	0.025 (0.007)	0.41 (0.09)	0.49 (0.06)
	FGL	0.168	0.009	0.094 (0.016)	0.13 (0.03)	0.60 (0.06)	0.051 (0.010)	0.25 (0.05)	0.62 (0.06)
	FGL (eBIC)	0.508	0.002	0.000 (0.000)	-	-	0.000 (0.000)	-	-
	GGL	0.167	0.012	0.091 (0.018)	0.14 (0.03)	0.59 (0.06)	0.049 (0.012)	0.26 (0.06)	0.61 (0.06)
	SSJGL	-	-	0.003 (0.001)	0.88 (0.09)	0.12 (0.02)	0.003 (0.001)	0.93 (0.07)	0.12 (0.02)
	stabJGL	0.218	0.088	0.011 (0.003)	0.60 (0.07)	0.33 (0.05)	0.008 (0.001)	0.76 (0.08)	0.30 (0.05)
0 %	Glasso	0.238	-	0.022 (0.009)	0.38 (0.10)	0.37 (0.08)	0.025 (0.007)	0.43 (0.08)	0.51 (0.07)
	FGL	0.167	0.006	0.098 (0.015)	0.13 (0.02)	0.62 (0.06)	0.053 (0.009)	0.26 (0.04)	0.66 (0.06)
	FGL (eBIC)	0.520	0.002	0.000 (0.000)	-	-	0.000 (0.000)	-	-
	GGL	0.167	0.008	0.095 (0.018)	0.13 (0.03)	0.61 (0.06)	0.051 (0.011)	0.27 (0.05)	0.65 (0.07)
	SSJGL	-	-	0.002 (0.000)	0.79 (0.11)	0.09 (0.02)	0.002 (0.000)	0.90 (0.08)	0.10 (0.02)
	stabJGL	0.218	0.088	0.010 (0.002)	0.58 (0.07)	0.29 (0.05)	0.007 (0.001)	0.76 (0.08)	0.26 (0.04)

Table S5: Performance of the different graph reconstruction methods in simulations, reconstructing graphs with $p = 300$ nodes from $K = 2$ classes with various similarity of the true graph structures. The methods included are the graphical lasso (Glasso), the fused joint graphical lasso tuned by the AIC (FGL) and by the extended BIC (eBIC), the group joint graphical lasso (GGL) and stabJGL. The similarity (percentage of edges that are in common) of the graphs is shown. The results are averaged over $N = 100$ simulations and shows the sparsity, precision, and recall of each of the $K = 2$ estimated graphs. The corresponding standard deviations are shown as well. The graphs are reconstructed from $n_1 = 150$ and $n_2 = 200$ observations. All graphs have sparsity 0.007. The average selected values of the penalty parameters λ_1 and λ_2 for the relevant methods is shown as well.

				$n_1 = 150$			$n_2 = 200$		
Similarity	Method	λ_1	λ_2	Sparsity	Precision	Recall	Sparsity	Precision	Recall
100 %	Glasso	0.195	-	0.009 (0.003)	0.26 (0.07)	0.30 (0.04)	0.006 (0.002)	0.39 (0.09)	0.31 (0.04)
	FGL	0.166	0.022	0.012 (0.005)	0.25 (0.11)	0.39 (0.03)	0.007 (0.003)	0.41 (0.14)	0.37 (0.03)
	FGL (eBIC)	0.453	0.001	0.000 (0.000)	-	-	0.000 (0.000)	-	-
	GGL	0.166	0.004	0.020 (0.003)	0.14 (0.02)	0.40 (0.03)	0.011 (0.002)	0.24 (0.04)	0.39 (0.03)
	stabJGL	0.166	0.098	0.003 (0.000)	0.69 (0.03)	0.35 (0.02)	0.003 (0.000)	0.72 (0.04)	0.35 (0.02)
80 %	Glasso	0.195	-	0.008 (0.003)	0.27 (0.07)	0.30 (0.04)	0.006 (0.003)	0.39 (0.14)	0.30 (0.06)
	FGL	0.166	0.012	0.016 (0.005)	0.18 (0.06)	0.39 (0.03)	0.008 (0.003)	0.32 (0.10)	0.37 (0.03)
	FGL (eBIC)	0.442	0.002	0.000 (0.000)	-	-	0.000 (0.000)	-	-
	GGL	0.166	0.005	0.019 (0.004)	0.14 (0.03)	0.40 (0.03)	0.010 (0.002)	0.26 (0.05)	0.37 (0.03)
	stabJGL	0.166	0.093	0.003 (0.000)	0.69 (0.04)	0.32 (0.02)	0.003 (0.000)	0.72 (0.04)	0.30 (0.02)
60 %	Glasso	0.195	-	0.008 (0.003)	0.26 (0.07)	0.30 (0.04)	0.006 (0.002)	0.38 (0.11)	0.31 (0.05)
	FGL	0.166	0.010	0.017 (0.005)	0.17 (0.05)	0.40 (0.03)	0.009 (0.003)	0.30 (0.08)	0.37 (0.03)
	FGL (eBIC)	0.449	0.002	0.000 (0.000)	-	-	0.000 (0.000)	-	-
	GGL	0.166	0.005	0.020 (0.004)	0.14 (0.02)	0.40 (0.03)	0.011 (0.002)	0.25 (0.05)	0.38 (0.03)
	stabJGL	0.166	0.091	0.003 (0.000)	0.65 (0.04)	0.29 (0.02)	0.003 (0.000)	0.70 (0.04)	0.28 (0.02)
40 %	Glasso	0.196	-	0.009 (0.003)	0.26 (0.08)	0.30 (0.05)	0.006 (0.003)	0.41 (0.13)	0.29 (0.06)
	FGL	0.166	0.007	0.018 (0.004)	0.15 (0.04)	0.40 (0.03)	0.009 (0.002)	0.27 (0.07)	0.36 (0.03)
	FGL (eBIC)	0.446	0.002	0.000 (0.000)	-	-	0.000 (0.000)	-	-
	GGL	0.166	0.004	0.020 (0.004)	0.14 (0.03)	0.40 (0.03)	0.010 (0.002)	0.25 (0.05)	0.37 (0.03)
	stabJGL	0.166	0.091	0.003 (0.000)	0.64 (0.05)	0.25 (0.02)	0.002 (0.000)	0.68 (0.04)	0.23 (0.02)
20 %	Glasso	0.195	-	0.008 (0.003)	0.28 (0.08)	0.30 (0.04)	0.006 (0.003)	0.38 (0.13)	0.30 (0.06)
	FGL	0.166	0.006	0.019 (0.004)	0.14 (0.03)	0.39 (0.03)	0.010 (0.002)	0.26 (0.06)	0.37 (0.04)
	FGL (eBIC)	0.450	0.001	0.000 (0.000)	-	-	0.000 (0.000)	-	-
	GGL	0.166	0.004	0.020 (0.003)	0.14 (0.02)	0.40 (0.03)	0.010 (0.002)	0.25 (0.04)	0.37 (0.03)
	stabJGL	0.166	0.091	0.002 (0.000)	0.60 (0.05)	0.22 (0.02)	0.002 (0.000)	0.63 (0.05)	0.19 (0.02)
0 %	Glasso	0.198	-	0.008 (0.003)	0.27 (0.07)	0.30 (0.04)	0.005 (0.002)	0.50 (0.12)	0.37 (0.07)
	FGL	0.166	0.000	0.022 (0.001)	0.13 (0.01)	0.42 (0.02)	0.012 (0.001)	0.28 (0.02)	0.51 (0.03)
	FGL (eBIC)	0.462	0.002	0.000 (0.000)	-	-	0.000 (0.000)	-	-
	GGL	0.166	0.001	0.022 (0.002)	0.13 (0.01)	0.41 (0.02)	0.012 (0.001)	0.28 (0.02)	0.51 (0.03)
	stabJGL	0.166	0.077	0.003 (0.001)	0.48 (0.05)	0.20 (0.03)	0.002 (0.000)	0.62 (0.04)	0.21 (0.03)

Table S6: Performance of the different graph reconstruction methods in simulations, reconstructing graphs with $p = 1000$ nodes from $K = 2$ classes with various similarity of the true graph structures. The methods included are the graphical lasso (Glasso), the fused joint graphical lasso tuned by the AIC (FGL), the group joint graphical lasso (GGL) and stabJGL. The similarity (percentage of edges that are in common) of the graphs is shown. The results are averaged over $N = 10$ simulations and shows the sparsity, precision, and recall of each of the $K = 2$ estimated graphs. The corresponding standard deviations are shown as well. The graphs are reconstructed from $n_1 = n_2 = 500$ observations. All graphs have sparsity 0.002. The average selected values of the penalty parameters λ_1 and λ_2 for the relevant methods is shown as well.

				$n_1 = 500$			$n_2 = 500$		
Similarity	Method	λ_1	λ_2	Sparsity	Precision	Recall	Sparsity	Precision	Recall
100 %	Glasso	0.185	-	0.000 (0.000)	0.75 (0.17)	0.12 (0.02)	0.000 (0.000)	0.78 (0.19)	0.12 (0.03)
	FGL	0.114	0.026	0.003 (0.001)	0.30 (0.10)	0.39 (0.01)	0.003 (0.001)	0.29 (0.10)	0.39 (0.02)
	GGL	0.114	0.016	0.004 (0.001)	0.16 (0.02)	0.34 (0.03)	0.004 (0.001)	0.16 (0.02)	0.34 (0.03)
	stabJGL	0.166	0.061	0.000 (0.000)	0.99 (0.01)	0.11 (0.00)	0.000 (0.000)	0.99 (0.01)	0.11 (0.00)
80 %	Glasso	0.186	-	0.000 (0.000)	0.78 (0.20)	0.12 (0.03)	0.000 (0.000)	0.78 (0.21)	0.12 (0.03)
	FGL	0.114	0.023	0.003 (0.001)	0.24 (0.05)	0.36 (0.01)	0.003 (0.001)	0.24 (0.04)	0.37 (0.01)
	GGL	0.114	0.016	0.004 (0.001)	0.15 (0.03)	0.33 (0.02)	0.004 (0.001)	0.16 (0.04)	0.34 (0.02)
	stabJGL	0.166	0.034	0.000 (0.000)	0.95 (0.03)	0.11 (0.00)	0.000 (0.000)	0.96 (0.03)	0.11 (0.01)
60 %	Glasso	0.189	-	0.000 (0.000)	0.76 (0.18)	0.12 (0.02)	0.000 (0.000)	0.86 (0.16)	0.10 (0.03)
	FGL	0.114	0.019	0.004 (0.001)	0.19 (0.05)	0.34 (0.02)	0.004 (0.001)	0.19 (0.04)	0.35 (0.02)
	GGL	0.114	0.017	0.004 (0.001)	0.16 (0.03)	0.31 (0.03)	0.004 (0.001)	0.16 (0.03)	0.32 (0.03)
	stabJGL	0.166	0.027	0.000 (0.000)	0.92 (0.04)	0.11 (0.01)	0.000 (0.000)	0.93 (0.04)	0.11 (0.01)
40 %	Glasso	0.191	-	0.000 (0.000)	0.79 (0.18)	0.12 (0.03)	0.000 (0.000)	0.88 (0.14)	0.11 (0.02)
	FGL	0.114	0.017	0.004 (0.001)	0.17 (0.05)	0.34 (0.03)	0.004 (0.001)	0.17 (0.05)	0.34 (0.03)
	GGL	0.114	0.018	0.004 (0.001)	0.16 (0.03)	0.31 (0.03)	0.004 (0.001)	0.17 (0.03)	0.32 (0.03)
	stabJGL	0.166	0.022	0.000 (0.000)	0.87 (0.04)	0.12 (0.01)	0.000 (0.000)	0.90 (0.06)	0.11 (0.01)
20 %	Glasso	0.189	-	0.000 (0.000)	0.72 (0.20)	0.13 (0.03)	0.000 (0.000)	0.92 (0.04)	0.09 (0.01)
	FGL	0.114	0.014	0.005 (0.001)	0.13 (0.02)	0.35 (0.02)	0.005 (0.001)	0.14 (0.02)	0.34 (0.02)
	GGL	0.114	0.015	0.005 (0.001)	0.14 (0.02)	0.33 (0.02)	0.005 (0.001)	0.14 (0.02)	0.32 (0.02)
	stabJGL	0.166	0.021	0.000 (0.000)	0.85 (0.05)	0.12 (0.01)	0.000 (0.000)	0.86 (0.02)	0.11 (0.01)
0 %	Glasso	0.185	-	0.000 (0.000)	0.73 (0.17)	0.13 (0.03)	0.001 (0.000)	0.90 (0.09)	0.25 (0.08)
	FGL	0.114	0.005	0.009 (0.002)	0.09 (0.01)	0.38 (0.04)	0.009 (0.002)	0.16 (0.03)	0.70 (0.03)
	GGL	0.114	0.008	0.007 (0.002)	0.10 (0.01)	0.36 (0.04)	0.008 (0.002)	0.18 (0.03)	0.68 (0.03)
	stabJGL	0.166	0.011	0.000 (0.000)	0.74 (0.06)	0.13 (0.01)	0.001 (0.000)	0.88 (0.03)	0.30 (0.03)

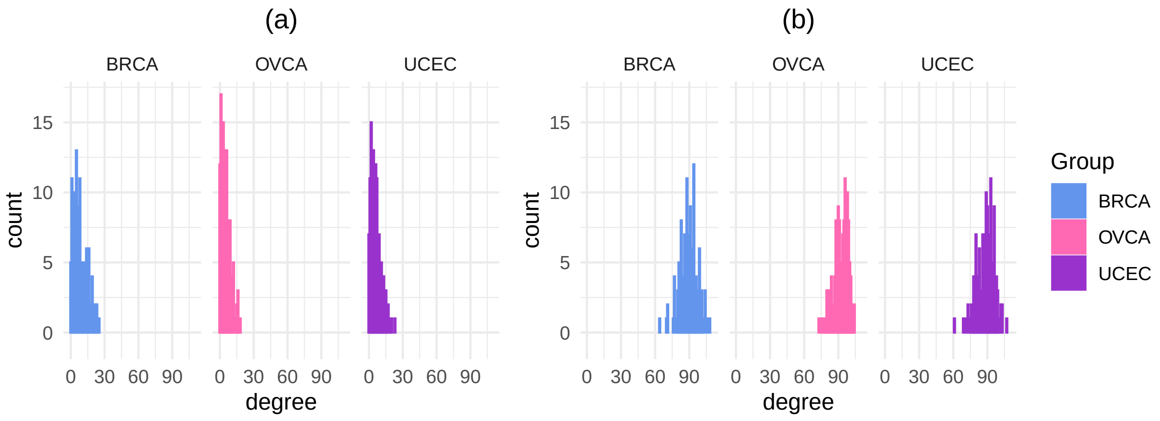

Figure S6: Histogram of the node degrees of the proteomic network of each tumor type, for the (a) stabJGL and (b) FGL networks.

Table S7: Performance of the different graph reconstruction methods in simulations, reconstructing graphs with $p = 100$ nodes from $K = 3$ networks with various similarity of the true graph structures. The methods included stabJGL with λ_1 selected before λ_2 (stabJGL), and stabJGL with λ_1 selected after λ_2 (stabJGL$_{\text{alt}}$). The similarity (percentage of edges that are in common) of the graphs is shown. The results are averaged over $N = 100$ simulations and shows the sparsity, precision, and recall of each of the $K = 3$ estimated graphs. The corresponding standard deviations are shown in parentheses. The graphs are reconstructed from $n_1 = 150$, $n_2 = 200$ and $n_3 = 300$ observations. All graphs have sparsity 0.02. The average selected values of the penalty parameters λ_1 and λ_2 for the methods is shown as well.

				$n_1 = 150$			$n_2 = 200$			$n_3 = 300$		
Similarity	Method	λ_1	λ_2	Sparsity	Precision	Recall	Sparsity	Precision	Recall	Sparsity	Precision	Recall
100 %	stabJGL	0.166	0.067	0.015 (0.001)	0.88 (0.05)	0.66 (0.04)	0.015 (0.001)	0.90 (0.04)	0.66 (0.04)	0.015 (0.001)	0.91 (0.03)	0.66 (0.04)
	stabJGL$_{\text{alt}}$	0.124	0.065	0.032 (0.007)	0.57 (0.10)	0.88 (0.11)	0.030 (0.007)	0.62 (0.13)	0.88 (0.11)	0.029 (0.007)	0.63 (0.14)	0.88 (0.11)
80 %	stabJGL	0.166	0.053	0.014 (0.002)	0.84 (0.08)	0.59 (0.04)	0.012 (0.001)	0.90 (0.04)	0.54 (0.04)	0.012 (0.001)	0.93 (0.03)	0.56 (0.04)
	stabJGL$_{\text{alt}}$	0.131	0.052	0.028 (0.009)	0.58 (0.13)	0.77 (0.12)	0.024 (0.008)	0.65 (0.17)	0.72 (0.11)	0.023 (0.008)	0.70 (0.17)	0.73 (0.12)
60 %	stabJGL	0.166	0.044	0.015 (0.003)	0.75 (0.09)	0.55 (0.05)	0.012 (0.001)	0.87 (0.05)	0.50 (0.04)	0.011 (0.001)	0.92 (0.04)	0.52 (0.04)
	stabJGL$_{\text{alt}}$	0.155	0.044	0.020 (0.008)	0.66 (0.13)	0.61 (0.10)	0.016 (0.007)	0.79 (0.16)	0.56 (0.09)	0.015 (0.006)	0.84 (0.16)	0.57 (0.09)
40 %	stabJGL	0.166	0.038	0.016 (0.003)	0.64 (0.09)	0.51 (0.04)	0.011 (0.001)	0.83 (0.06)	0.46 (0.04)	0.009 (0.001)	0.93 (0.03)	0.44 (0.04)
	stabJGL$_{\text{alt}}$	0.161	0.039	0.018 (0.006)	0.62 (0.09)	0.52 (0.08)	0.013 (0.005)	0.78 (0.12)	0.48 (0.07)	0.011 (0.004)	0.90 (0.11)	0.47 (0.08)
20 %	stabJGL	0.166	0.036	0.016 (0.003)	0.61 (0.08)	0.48 (0.05)	0.012 (0.002)	0.79 (0.07)	0.46 (0.05)	0.010 (0.001)	0.92 (0.04)	0.45 (0.04)
	stabJGL$_{\text{alt}}$	0.166	0.036	0.017 (0.003)	0.60 (0.09)	0.49 (0.05)	0.012 (0.002)	0.78 (0.07)	0.46 (0.05)	0.010 (0.001)	0.92 (0.04)	0.46 (0.04)
0 %	stabJGL	0.166	0.024	0.025 (0.006)	0.43 (0.07)	0.51 (0.05)	0.018 (0.003)	0.73 (0.07)	0.64 (0.07)	0.015 (0.002)	0.90 (0.04)	0.66 (0.08)
	stabJGL$_{\text{alt}}$	0.166	0.023	0.025 (0.005)	0.41 (0.06)	0.51 (0.05)	0.019 (0.003)	0.71 (0.07)	0.65 (0.06)	0.015 (0.002)	0.90 (0.04)	0.68 (0.07)

Table S8: Performance of the different graph reconstruction methods in simulations, reconstructing graphs with $p = 100$ nodes from $K = 3$ networks with various similarity of the true graph structures and with large random noise added. The methods included are Glasso, FGL and GGL tuned by AIC, FGL tuned by eBIC, SSJGL and stabJGL. The similarity (percentage of edges that are in common) of the graphs is shown. The results are averaged over $N = 100$ simulations and shows the sparsity, precision, and recall of each of the $K = 3$ estimated graphs. The corresponding standard deviations are shown in parentheses. The graphs are reconstructed from $n_1 = 150$, $n_2 = 200$ and $n_3 = 300$ observations. All graphs have sparsity 0.02. The average selected values of the penalty parameters λ_1 and λ_2 for the relevant methods is shown as well.

				$n_1 = 150$			$n_2 = 200$			$n_3 = 300$		
Similarity	Method	λ_1	λ_2	Sparsity	Precision	Recall	Sparsity	Precision	Recall	Sparsity	Precision	Recall
100 %	Glasso	0.202	-	0.018 (0.009)	0.18 (0.07)	0.14 (0.05)	0.009 (0.005)	0.33 (0.13)	0.13 (0.06)	0.004 (0.002)	0.61 (0.14)	0.12 (0.05)
	FGL	0.174	0.005	0.041 (0.022)	0.18 (0.19)	0.24 (0.11)	0.023 (0.015)	0.28 (0.20)	0.22 (0.10)	0.009 (0.007)	0.54 (0.20)	0.20 (0.10)
	GGL	0.176	0.006	0.040 (0.020)	0.15 (0.14)	0.23 (0.09)	0.022 (0.014)	0.26 (0.19)	0.21 (0.09)	0.008 (0.007)	0.53 (0.18)	0.18 (0.08)
	SSJGL	-	-	0.001 (0.001)	0.75 (0.34)	0.03 (0.02)	0.001 (0.001)	0.75 (0.34)	0.03 (0.02)	0.001 (0.001)	0.75 (0.34)	0.03 (0.02)
	stabJGL	0.166	0.068	0.004 (0.001)	0.88 (0.12)	0.17 (0.04)	0.004 (0.001)	0.92 (0.07)	0.17 (0.04)	0.004 (0.001)	0.93 (0.06)	0.17 (0.04)
80 %	Glasso	0.201	-	0.017 (0.008)	0.18 (0.06)	0.14 (0.05)	0.009 (0.004)	0.45 (0.13)	0.17 (0.06)	0.007 (0.002)	0.72 (0.11)	0.23 (0.05)
	FGL	0.153	0.006	0.056 (0.025)	0.12 (0.04)	0.31 (0.10	0.032 (0.018)	0.24 (0.09)	0.33 (0.09)	0.015 (0.009)	0.53 (0.15)	0.35 (0.09)
	GGL	0.165	0.002	0.050 (0.018)	0.11 (0.02)	0.26 (0.06)	0.027 (0.013)	0.23 (0.04)	0.30 (0.05)	0.012 (0.007)	0.55 (0.10)	0.31 (0.05)
	SSJGL	-	-	0.001 (0.001)	0.99 (0.06)	0.06 (0.03)	0.001 (0.001)	0.99 (0.06)	0.06 (0.03)	0.001 (0.001)	0.99 (0.06)	0.06 (0.03)
	stabJGL	0.166	0.074	0.004 (0.001)	0.92 (0.09)	0.20 (0.04)	0.004 (0.001)	0.94 (0.05)	0.20 (0.03)	0.004 (0.001)	0.96 (0.04)	0.20 (0.03)
60 %	Glasso	0.200	-	0.018 (0.007)	0.19 (0.06)	0.15 (0.05)	0.010 (0.005)	0.42 (0.14)	0.18 (0.06)	0.007 (0.003)	0.72 (0.13)	0.25 (0.06)
	FGL	0.153	0.005	0.059 (0.028)	0.11 (0.04)	0.30 (0.09)	0.033 (0.021)	0.22 (0.07)	0.32 (0.09)	0.017 (0.012)	0.50 (0.14)	0.36 (0.09)
	GGL	0.164	0.002	0.052 (0.021)	0.11 (0.03)	0.27 (0.07)	0.028 (0.017)	0.23 (0.06)	0.29 (0.07)	0.014 (0.009)	0.53 (0.10)	0.33 (0.07)
	SSJGL	-	-	0.001 (0.001)	0.99 (0.07)	0.05 (0.03)	0.001 (0.001)	0.98 (0.08)	0.05 (0.03)	0.001 (0.001)	0.99 (0.07)	0.05 (0.03)
	stabJGL	0.166	0.071	0.004 (0.001)	0.89 (0.12)	0.17 (0.03)	0.004 (0.001)	0.94 (0.06)	0.17 (0.03)	0.004 (0.001)	0.95 (0.06)	0.17 (0.03)
40 %	Glasso	0.198	-	0.018 (0.008)	0.17 (0.07)	0.14 (0.05)	0.011 (0.004)	0.39 (0.11)	0.20 (0.05)	0.006 (0.002)	0.72 (0.14)	0.19 (0.06)
	FGL	0.161	0.003	0.052 (0.021)	0.11 (0.03)	0.26 (0.08)	0.029 (0.015)	0.23 (0.06)	0.30 (0.07)	0.012 (0.008)	0.53 (0.13)	0.28 (0.07)
	GGL	0.166	0.003	0.047 (0.013)	0.11 (0.02)	0.24 (0.06)	0.025 (0.009)	0.23 (0.04)	0.29 (0.05)	0.010 (0.005)	0.55 (0.09)	0.27 (0.05)
	SSJGL	-	-	0.002 (0.003)	0.72 (0.35)	0.04 (0.04)	0.002 (0.003)	0.76 (0.33)	0.04 (0.04)	0.002 (0.003)	0.75 (0.35)	0.04 (0.03)
	stabJGL	0.166	0.081	0.002 (0.001)	0.84 (0.14)	0.08 (0.03)	0.002 (0.001)	0.87 (0.12)	0.08 (0.03)	0.002 (0.001)	0.88 (0.10)	0.08 (0.02)
20 %	Glasso	0.200	-	0.019 (0.008)	0.19 (0.06)	0.16 (0.05)	0.010 (0.004)	0.45 (0.12)	0.22 (0.06)	0.007 (0.002)	0.73 (0.11)	0.24 (0.05)
	FGL	0.149	0.005	0.066 (0.031)	0.10 (0.03)	0.31 (0.08)	0.040 (0.023)	0.22 (0.07)	0.37 (0.08)	0.019 (0.012)	0.46 (0.14)	0.36 (0.08)
	GGL	0.162	0.002	0.055 (0.027)	0.11 (0.03)	0.28 (0.06)	0.032 (0.020)	0.24 (0.05)	0.34 (0.07)	0.015 (0.011)	0.52 (0.11)	0.33 (0.07)
	SSJGL	-	-	0.001 (0.002)	0.79 (0.32)	0.02 (0.02)	0.001 (0.002)	0.88 (0.25)	0.03 (0.03)	0.001 (0.002)	0.88 (0.27)	0.03 (0.03)
	stabJGL	0.166	0.077	0.002 (0.001)	0.79 (0.15)	0.08 (0.03)	0.002 (0.001)	0.86 (0.10)	0.10 (0.04)	0.002 (0.001)	0.90 (0.10)	0.09 (0.03)
0 %	Glasso	0.201	-	0.017 (0.007)	0.18 (0.06)	0.14 (0.05)	0.008 (0.005)	0.39 (0.13)	0.14 (0.06)	0.005 (0.003)	0.69 (0.14)	0.15 (0.07)
	FGL	0.170	0.003	0.042 (0.017)	0.13 (0.10)	0.23 (0.08)	0.021 (0.019)	0.27 (0.13)	0.24 (0.08)	0.009 (0.005)	0.56 (0.15)	0.22 (0.09)
	GGL	0.170	0.006	0.040 (0.014)	0.13 (0.05)	0.23 (0.07)	0.020 (0.008)	0.26 (0.10)	0.23 (0.07)	0.008 (0.003)	0.58 (0.13)	0.21 (0.08)
	SSJGL	-	-	0.003 (0.003)	0.33 (0.34)	0.03 (0.02)	0.003 (0.003)	0.41 (0.36)	0.03 (0.03)	0.003 (0.003)	0.37 (0.36)	0.03 (0.03)
	stabJGL	0.166	0.083	0.000 (0.000)	0.71 (0.35)	0.02 (0.01)	0.000 (0.000)	0.79 (0.30)	0.02 (0.01)	0.000 (0.000)	0.71 (0.34)	0.01 (0.01)

Table S9: The genes with node degree larger than the 90th percentile in the respective stabJGL networks of the different tumor types. The genes that have node degree in the upper 10% in all three tumor types are marked in bold. The genes that only have node degree in the upper 10% in one tumor type are marked in red.

BRCA			UCEC			OVCA		
Protein	Gene	Degree	Protein	Gene	Degree	Protein	Gene	Degree
mTOR	*MTOR*	25	**14-3-3-epsilon**	*YWHAE*	23	Bak	*BAK1*	18
**14-3-3-epsilon**	*YWHAE*	24	CD31	*PECAM1*	20	MRE11	*MRE11A*	17
EGFR	*EGFR*	23	MRE11	*MRE11A*	19	**EGFR-pY1173**	*EGFR*	16
Chk1	*CHEK1*	23	GSK-3-alpha-beta-pS21S9	*GSK3A*	17	**Bid**	*BID*	16
Tuberin	*TSC2*	22	**EGFR-pY1173**	*EGFR*	17	**14-3-3-epsilon**	*YWHAE*	16
**EGFR-pY1173**	*EGFR*	22	p38-pT180-Y182	*MAPK14*	16	**Stathmin**	*STMN1*	15
**Stathmin**	*STMN1*	21	**Stathmin**	*STMN1*	15	MAPK-pT202-Y204	*MAPK1*	15
c-KIT	*KIT*	21	LKB1	*STK11*	15	GSK-3-alpha-beta-pS21S9	*GSK3A*	13
Ku80	*XRCC5*	20	**Bid**	*BID*	15	SMAD4	*SMAD4*	12
S6	*RPS6*	19	MIG6	*ERRFI1*	14	MIG6	*ERRFI1*	12
Hsp70	*HSPA1A*	19	MAPK-pT202-Y204	*MAPK1*	14	Cyclin D1	*CCND1*	12
Collagen VI	*COL6A1*	19	c-Met-pY1235	*MET*	14	Chk1	*CHEK1*	12
**Bid**	*BID*	19				c-Met-pY1235	*MET*	12

Table S10: The genes with node degree larger than the 90th percentile in the respective FGL networks of the different tumor types. The genes that have node degree in the upper 10% in all three tumor types are marked in bold. The genes that only have node degree in the upper 10% in one tumor type are marked in red.

BRCA			UCEC			OVCA		
Protein	Gene	Degree	Protein	Gene	Degree	Protein	Gene	Degree
NF-kB-p65-pS536	*NFKB1*	108	Src	*SRC*	107	SYK	*SYK*	104
AR	*AR*	106	PEA15	*PEA-15*	103	p70-S6K	*RPS6KB1*	104
PAI-1	*SERPINE1*	104	JNK2	*MAPK9*	103	mTOR-pS2448	*MTOR*	103
XRCC1	*XRCC1*	104	HER3	*ERBB3*	102	ER-alpha-pS118	*ESR1*	102
Cyclin E1	*CCNE1*	104	c-Raf	*RAF1*	102	ATM	*ATM*	102
S6	*RPS6*	102	VEGFR2	*KDR*	100	STAT5-alpha	*STAT5A*	101
RAD50	*RAD50*	101	p53	*TP53*	99	PCNA	*PCNA*	101
MEK1	*MAP2K1*	101	GSK-3-alpha-beta	*GSK3A*	99	MEK1	*MAP2K1*	101
INPP4B	*INPP4B*	101	AMPK-pT172	*PRKAA1*	99	53BP1	*TP53BP1*	101
MIG6	*ERRFI1*	100	Tuberin	*TSC2*	98			
HER3	*ERBB3*	100	p38-MAPK	*MAPK14*	98			
			p27	*CDKN1B*	98			
			IGFBP2	*IGFBP2*	98			
